# Histone Variant H2A.Z Enhances Histone and Nucleosome Dynamics

**DOI:** 10.1016/j.mcpro.2026.101518

**Published:** 2026-01-29

**Authors:** Juliana Kikumoto Dias, Prabavi Shayana Dias, Rakhat Alakenova, Charles Mariasoosai, Claudia Claridy, Sameeha Gazi, Hedieh Torabifard, Sheena D'Arcy

**Affiliations:** Department of Chemistry & Biochemistry, The University of Texas at Dallas, Richardson, Texas, USA

## Abstract

Interchanging canonical histone H2A with variant H2A.Z in chromatin complexes is vital for the proper regulation of transcription, DNA damage repair, and centromere maintenance. However, the physical mechanisms underlying functional differences between H2A and H2A.Z complexes are unclear. Human H2A and H2A.Z exhibit high sequence and structural conservation, with subtle differences in the H2A DNA-binding loops. In this study, we employ hydrogen-deuterium exchange coupled with mass spectrometry and molecular dynamics simulation to investigate the differences in solution behavior between human H2A-H2B and H2A.Z-H2B. We demonstrate that replacing H2A with H2A.Z enhances the dynamics of the refolded histone heterodimer, whether it is in nucleosomes, in complex with H3-H4, or alone in solution. In all situations, enhanced dynamics are observed for H2B, suggesting altered interaction with H2A.Z and DNA. Parallel comparisons of H2A-H2B orthologs between humans and frogs reveal fewer differences in dynamics. Our findings provide mechanistic insights into the function of histone variants and reveal how differences in dynamics may underlie functional differences between structurally similar proteins.

## Highlights

• Histone sequences and structures are highly conserved among variants and homologs.
• We use modified HDX-MS to compare histone homologs distinguished by stable isotopes.
• Replacing H2A with H2A.Z in histone complexes enhances the dynamics of H2B.
• MD simulations show fewer H-bonds in nucleosomes with H2A.Z in place of H2A.

## Graphical Abstract

**Figure:**
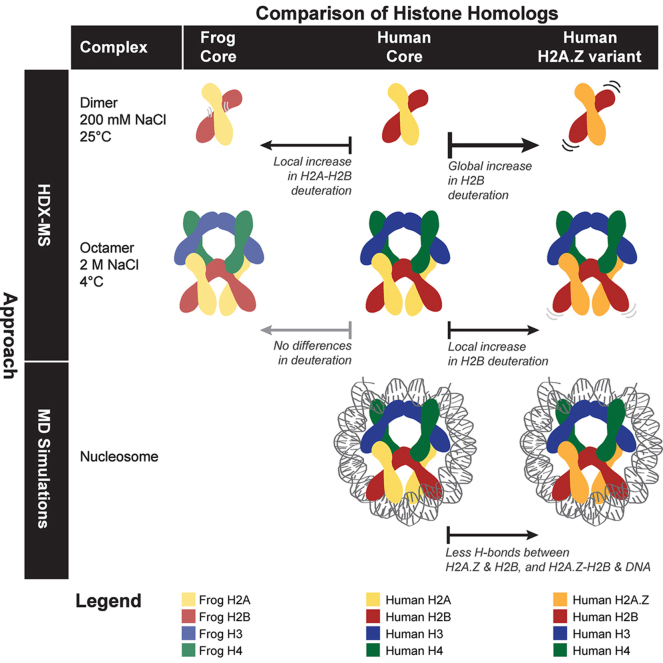

## In Brief

The sequence and structure of the histone H2A-H2B heterodimer are highly conserved among eukaryotic homologs, such as frogs and humans, as well as functionally distinct variants, such as H2A.Z-H2B. Using hydrogen-deuterium exchange mass spectrometry and molecular dynamics simulations, we show that amino acid substitutions, even those seemingly conservative, can alter the solution dynamics of the histone heterodimer when it is alone and, in some cases, when it is bound to the histone (H3-H4)_2_ tetramer or in the nucleosome.

Nucleosomes are the fundamental building blocks of chromatin, responsible for packaging DNA within the eukaryotic nucleus. Each nucleosome consists of a histone protein octamer comprising two copies of each core histone (H2A, H2B, H3, and H4) around which approximately 147 base pairs of double-stranded DNA wrap (1). This highly organized histone–DNA complex plays a pivotal role in controlling access to the DNA, thereby regulating transcription, DNA repair, and DNA replication. Histones are among the most conserved proteins, with orthologous nucleosomes showing almost no structural differences (1, 2, 3, 4, 5). Most *in vitro* studies use *Xenopus laevis* (*Xl*) histones; their purification and nucleosome reconstitution protocols are well-established (6). Histones H2A-H2B and H3-H4 differ between *Xl* and *Homo sapiens* (*Hs*) at only 16 out of 251 and 2 out of 237 residues, respectively. Here, we use hydrogen-deuterium exchange coupled with mass spectrometry (HDX-MS) to determine whether a few amino acid substitutions can alter histone dynamics across various chromatin complexes. We hypothesized that differences in protein dynamics are important for protein families with high sequence and/or structural conservation.

Alongside core histones, eukaryotes have paralogous histone variants that can replace their canonical counterparts (7, 8). Histone variants play an important regulatory role by spatially and temporally enhancing chromatin's structural and functional diversity. Histone variant exchange by histone chaperones is independent of DNA replication and can occur throughout the cell cycle (9). Most histone variants exhibit high structural conservation within the three helices (α1, α2, and α3) and two loops (L1 and L2) that comprise the histone fold. Some variants have only a few amino acid substitutions, while others have highly diverged sequences. We have chosen to study the human histone variant H2A.Z as it is a versatile variant that functions in euchromatin and heterochromatin and plays critical roles in transcription, DNA damage repair, and centromere maintenance (10, 11, 12, 13).

Aberrant *Hs*H2A.Z function is linked to neurological disorders, metabolic syndromes, and heart disease (14). The *Hs*H2A.Z gene originated by duplication and maintains 60% sequence similarity with *Hs*H2A (15). Crystallography shows the structural conservation of human H2A and H2A.Z heterodimers with H2B, and of the nucleosomes with zero, one, or two copies of H2A replaced by H2A.Z (16, 17). Subtle differences occur only in the L1 DNA-binding loop, potentially accounting for the enhanced mobility of H2A.Z nucleosomes (16). H2A.Z octamers can slide along DNA and facilitate the DNA binding of regulatory factors, including RNA polymerase II, at transcriptional start sites (18, 19). We sought to further investigate the physical basis of histone variant function by studying how H2A.Z incorporation affects the dynamics of chromatin complexes using HDX-MS and molecular dynamics (MD) simulations.

HDX-MS measures the rate of deuterium exchange into the backbone amide groups of a protein (20). This rate is influenced by the stability of the hydrogen bond formed by the backbone amide group, provided other variables (such as exchange time, pH, temperature, and amino acid sequence) are tightly controlled (21, 22). Hydrogen bond stability is a direct readout of protein dynamics on the second-to-minute timescale and is influenced by the conformational ensemble and its interactions (23). We implement a non-standard HDX-MS workflow that uses histones prepared with Nitrogen-15 (^15^N) or Carbon-13 (^13^C) stable isotopes, alongside those with natural isotope abundance (hereafter, wild-type) (24). In a single sample, we monitor the exchange of three histone complexes, as ^15^N, ^13^C, and wild-type peptide spectra do not overlap. Compared to the standard HDX-MS workflow, the number of samples is reduced threefold, and exchange conditions are identical rather than just matched. We use ^15^N for core *Xl* samples, ^13^C for core *Hs* samples, and wild-type for variant *Hs* samples.

We directly compare the dynamics of core *Hs* histones to those from *Xl* or those containing histone variant H2A.Z. We measure dynamics using HDX-MS of histone heterodimers and histone octamers. The octamers are a surrogate for the nucleosome and are stabilized by low temperature and high ionic strength (25). In silico MD simulations reinforce the relevance of our experimental results to the nucleosome. Overall, we observe similar exchange between *Hs* and *Xl* histones in the dimer or octamer, suggesting that their few amino acid differences have little to no effect on protein dynamics. We observe more significant differences between core and variant histone complexes, with the incorporation of H2A.Z enhancing the dynamics of H2B within the histone dimer, the octamer, and the nucleosome. Dynamic changes occur around the H2B L2 loop, which can interact with H2A and DNA. Our data reveal that the H2A.Z histone variant profoundly impacts the stability of its H2B heterodimer partner, reducing DNA-binding interactions within nucleosomes. We provide a deeper understanding of the physical basis of histone variant function by highlighting a role for dynamics.

## Experimental Procedures

### Nucleosome Reconstitution Assays

Histone dimers and Widom 601 DNA were purified, and histone octamers and nucleosomes were assembled using established protocols (6, 26). For sequential histone deposition, (H3-H4)_2_ was titrated at 0.6, 1.0, and 1.4 M ratios against 1 μM DNA at 2 M NaCl. Salt was reduced to 200 mM via dialysis over 60 h using a Gilson Minipuls 3 and PlusOne dialysis tubes. H2A-H2B was titrated at 1.6, 2, and 2.4 M ratios against 1 μM tetrasome at 200 mM NaCl. For octamer deposition, the histone octamer was titrated at 0.6, 1, and 1.4 M ratios against 1 μM DNA at 2 M NaCl. Salt was reduced to 200 mM via dialysis over 60 h using a Gilson Minipuls 3 and PlusOne dialysis tubes. All molar ratios were analyzed at each step using 5% (w/v) acrylamide native-PAGE stained with EtBr. In addition to NaCl, the dialysis buffer contained 20 mM TRIS-HCl pH 7.5, 1 mM EDTA, and 1 mM DTT.

### HDX-MS

Histones with ^15^N and ^13^C isotopes were expressed in M9 minimal media supplemented with ^15^N-ammonium chloride or ^13^C-glucose, respectively, as described elsewhere (24). Using established protocols, ^15^N, ^13^C, and wild-type histones were purified, refolded, and reconstituted into octamers (6). Here, wild-type refers to histones with their natural isotope abundances. Samples were dialyzed into 20 mM TRIS-HCl pH 7.5, 1 mM EDTA, 1 mM DTT, and 200 mM or 2 M NaCl. One experiment (Experiment A) contained a single mixture at 200 mM NaCl and 25 °C of ^13^C *Hs*H2A-H2B, ^15^N *Xl*H2A-H2B, and wild-type *Hs*H2A.Z-H2B. A second experiment (Experiment B) contained three mixtures at 2 M NaCl and 4 °C; one with ^13^C *Hs*H2A-H2B, ^15^N *Xl*H2A-H2B, and wild-type *Hs*H2A.Z-H2B, one with ^13^C *Hs*(H3-H4)_2_, ^15^N *Xl*(H3-H4)_2_, and wild-type *Hs*(H3-H4)_2_, and one with ^13^C *Hs* core octamer, ^15^N *Xl* core octamer, and wild-type *Hs*H2A.Z octamer. The three mixtures were handled in parallel. For both experiments, the mixtures were equilibrated for at least 10 min before diluting with deuterated buffer to achieve a final concentration of 5 μM for each sample and an 87% (v/v) D_2_O solution. Non-deuterated controls were prepared similarly but with a non-deuterated buffer.

The exchange was minimized after 10^1^, 10^2^, 10^3^, and 10^4^ s by diluting 10 μl of each mixture 1:9 with a cooled buffer and quenching solution at a 4:5 (v/v) ratio, resulting in a pH of 2.6. The quench solution was 1.6 M GuCl, 0.8% (v/v) formic acid. Samples were flash-frozen in liquid nitrogen for storage at −80 °C. LC/MS was performed using the Waters HDX manager and SYNAPT G2-Si Q-Tof system. We analyzed four technical replicates, and the samples were injected in random order. Samples were digested online by *Sus scrofa* Pepsin A (Waters Enzymate BEH) for 3 min at 25 μl/min and 15 °C, and then the peptides were trapped on a C4 column (Waters Acquity UPLC Protein BEH) for 3 min at 200 μl/min at 1 °C. Peptides were separated over a C18 (Waters Acquity UPLC BEH) column in 0.1% (v/v) formic acid and eluted with a 3 to 40% linear gradient of 0.1% (v/v) formic acid in acetonitrile for 12 min at 40 μl/min and 1 °C.

All mass spectrometry data were obtained by operating in positive-ion mode, utilizing high-definition mass spectrometry HDMS^E^ mode. HDMS^E^ was specifically used to capture low-energy (at 6 V) and high-energy (with a ramping voltage range of 22–44 V) peptide fragmentation data to identify peptides in non-deuterated controls. HDMS^E^ mode was used for deuterated samples to collect low-energy ion data. In all cases, the samples were acquired in resolution mode. Key instrument parameters include a capillary voltage set at 2.8 kV for the sample sprayer, a desolvation gas flow rate of 650 L/h at 175 °C, a source temperature of 80 °C, and flow cone and nebulizer gas rates of 90 L/h and 6.5 bar, respectively. Additionally, the sampling cone and source offset were set to 30 V. Data acquisition was performed with a scan time of 0.4 s over a mass-to-charge ratio (m/z) range of 100 to 2000. Mass correction was implemented using [Glu1]-fibrinopeptide B as a reference mass.

Raw data from non-deuterated samples were processed in PLGS (Waters Protein Lynx Global Server 3.0.2). The database contained *S. scrofa* pepsin A and the corresponding *Xl* or *Hs* histone proteins. The minimum requirement for fragment ion matches per peptide was set at 3, and allowances were made for methionine oxidation. The data processing parameters included low and elevated energy thresholds, specifically 250 and 50 counts, respectively, and an overall intensity threshold of 750 counts. In the case of deuterated data, analysis was conducted using DynamX 3.0, with 5000 minimum intensity, 0.3 products per amino acid, one consecutive product, and an error of 20 ppm. The file threshold was 2 out of 4. Data was manually curated and not corrected for back exchange or normalized. The D-uptake and ΔHDX values reported use the measured D-uptake value or the difference between two D-uptake values and calculate them as a percentage of the theoretical maximum deuteration of each peptide. The theoretical maximum deuteration was calculated as follows: (number of residues in peptide – 1 for N-terminal residue – the number of prolines not at the N-terminal position). The processing and analysis of ^15^N and ^13^C peptides were done as described elsewhere (24). Assigned peptides are listed in Supplemental Table S1, and all raw files have been deposited into ProteomeXchange via the PRIDE partner repository (PXD062956) (27, 28). Structure images were made using PyMOL 3.0.2 (29).

### MD Simulation

Crystal structures of the human core and double-copy H2A.Z variant nucleosome were obtained from the RCSB Protein Data Bank (PDB: 2CV5 (30) and 3WA9 (30)). CHARMM-GUI (31) was used to construct the systems, maintaining pH 7.5. To best approximate physiological conditions, NH_3_^+^/COO^-^ capping groups were applied to the histone termini. Each system was neutralized with counterions (Na^+^ and Cl^−^, 200 mM) and solvated in a truncated rectangular periodic water box using the TIP3P water model (32), with a 12 Å buffer from the solute. MD simulations were performed using the AMBER 20 package (33) with the AMBER ff19SB force field (34) for protein structures and OL15 for DNA (35). Periodic boundary conditions were applied throughout the simulations, and a 9.0 Å cutoff was set for non-bonded interactions. The SHAKE algorithm (36) was used to constrain all bonds involving hydrogen atoms. A two-step energy minimization procedure was carried out. First, 5000 steps of steepest descent minimization were performed with the protein and nucleosome restrained by a harmonic force constant of 20 kcal/mol/Å, allowing only the water and ions to relax. All restraints were removed in the second minimization step, enabling the entire system to relax. Following minimization, the systems were gradually heated to 303.15 K using Langevin dynamics (37) in the NVT ensemble. The heating process was conducted in three stages: 1. the protein was restrained with a 20 kcal/mol/Å force constant for 2 ns, 2. restraints were reduced to 10 kcal/mol/Å for an additional 3 ns, and 3. further reduction to 5 kcal/mol/Å was applied for 5 ns. Equilibration was then carried out under NPT conditions for a total of 25 ns. Initially, harmonic restraints of 5.0 kcal/mol/Å were applied to the protein for 5 ns, followed by a reduction to 2.0 kcal/mol/Å for 10 ns. The final 10 ns of equilibration were conducted without restraints to ensure system stability. For production runs, 450 ns of MD simulations were performed under NPT conditions for each system, with three independent replicates conducted for statistical robustness. A time step of 2 fs was used, and the temperature was maintained at 303.15 K using a Langevin thermostat (37), while pressure was regulated at 1 atm with a Berendsen barostat (38).

### Trajectory Analysis

All analyses were performed using the cpptraj program from the AMBER20 software package (39). Since each nucleosome contains two copies of H2A-H2B or H2A.Z-H2B, we analyzed both copies, treating them as six independent trials. The root-mean-square deviation (RMSD) of backbone atoms and root-mean-square fluctuation (RMSF) of all residues were calculated to assess the stability and flexibility of the systems, respectively. Solvent-accessible surface area (SASA) calculations were performed using the SASA tool of GROMACS v2023.3 (40) to evaluate solvent exposure of the nucleosome model. A hydrogen bond analysis was conducted to identify key stabilizing interactions for both copies within the nucleosome, particularly between H2.A/H2A.Z, H2B, and DNA. Plots were created using the matplotlib Python library, and molecules were represented using PyMOL v3.1.1 (29). MD simulation input files, representative trajectories, and analysis scripts are uploaded to Zenodo (https://doi.org/10.5281/zenodo.15151314).

## Results

### Nucleosome Assembly By-Products Differ Between Histone Homologs

**Fig. 1:**
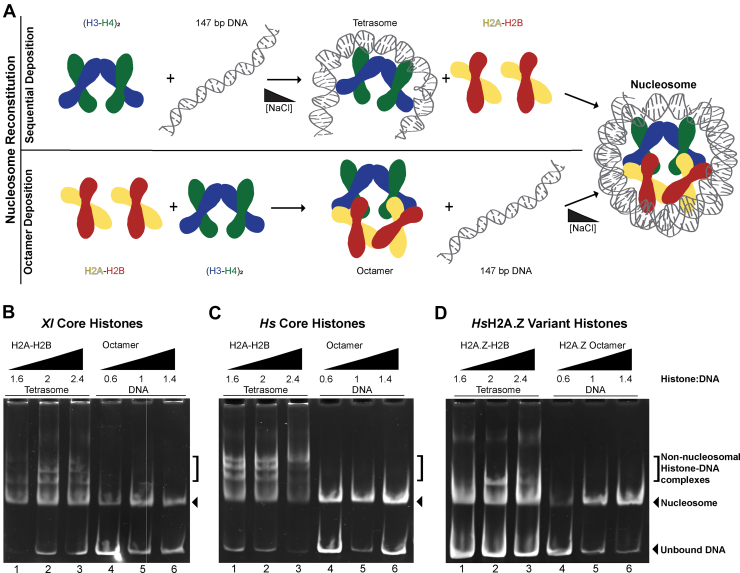
**Comparison of nucleosome assembly with H2A-H2B homologs.***A*, schematic of the two methods used for nucleosome reconstitution. *Top*: Sequential histone deposition *via* a tetrasome intermediate. (H3-H4)_2_ was deposited onto DNA using NaCl-gradient dialysis. *Bottom*: Octamer deposition using a purified histone octamer. Octamer was deposited onto DNA using NaCl-gradient dialysis. *B–D*, nucleosome assembly assays for core *Xl* histones, core *Hs* histones, and H2A.Z variant *Hs* histones. H2A-H2B was titrated against tetrasome at 1.6, 2.0, and 2.4 M ratios (lanes 1–3); histone octamer was titrated against DNA at 0.6, 1.0, and 1.4 M ratios (lanes 4–6). Native gels were 5% (w/v) acrylamide and stained with EtBr.

To compare the solution behavior of various H2A-H2B homologs, we first compared their nucleosome formation using native PAGE (Fig. 1). We reconstituted nucleosomes using sequential deposition of the (H3-H4)_2_ tetramer followed by two copies of H2A-H2B dimer, or by deposition of the histone octamer pre-purified and stabilized by high salt. Both deposition protocols use gradient dialysis from high salt (2 M NaCl) to low salt (200 mM NaCl) to regulate the interaction between histones and the 146 bp Widom 601 DNA positioning sequence at different steps (Fig. 1*A*). Native PAGE showed that as expected all histone types were successfully assembled into nucleosomes via octamer deposition (Fig. 1, *B*–*D*, lanes 4–6). We tested the deposition of octamer with all *Xl* core histones (Fig. 1*B*), *Hs* core histones (Fig. 1*C*), and *Hs* core histones with H2A replaced by variant H2A.Z (Fig. 1*D*). We added 0.6, 1, and 1.4 M equivalents of octamer to DNA. We observed nucleosomes with minimal by-products in all cases.

Sequential deposition also resulted in nucleosome formation for all H2A-H2B homologs, although by-products were observed (Fig. 1, *B*–*D*, lanes 1–3). The appearance of by-products reflects the ability of the H2A-H2B homolog to bind DNA as the histones are mixed with unbound DNA at low salt (Fig. 1*A*). The unbound DNA can be excess free DNA in the tetrasome sample, as well as the DNA that remains available for binding either side of the (H3-H4)_2_ tetramer, or even in a complete nucleosome. A H2A-H2B homolog with a tight affinity for DNA will tend to bind DNA in all these unbound contexts and thus lead to a larger number of by-products. Conversely, a H2A-H2B homolog with a weak affinity for DNA will bind mainly in a nucleosome context with both histone-DNA and histone-histone interactions and not have as many by-products. We added 1.2, 2.0, and 2.4 M equivalents of H2A-H2B to the tetrasome and observed distinct patterns of by-products for each H2A-H2B homolog. *Xl* and *Hs* core H2A-H2B (Fig. 1, *B* and *C*) had more by-products than *Hs* variant H2A.Z-H2B (Fig. 1*D*). The appearance of fewer by-products for H2A.Z-H2B than H2A-H2B thus suggests the variant has a comparatively weaker affinity for DNA. The distinct behavior of the H2A-H2B homologs in nucleosome reconstitution via sequential deposition prompted further analysis of the histone dimer and octamer using HDX-MS.

### HDX-MS of Homologous Histone Complexes

**Fig. 2:**
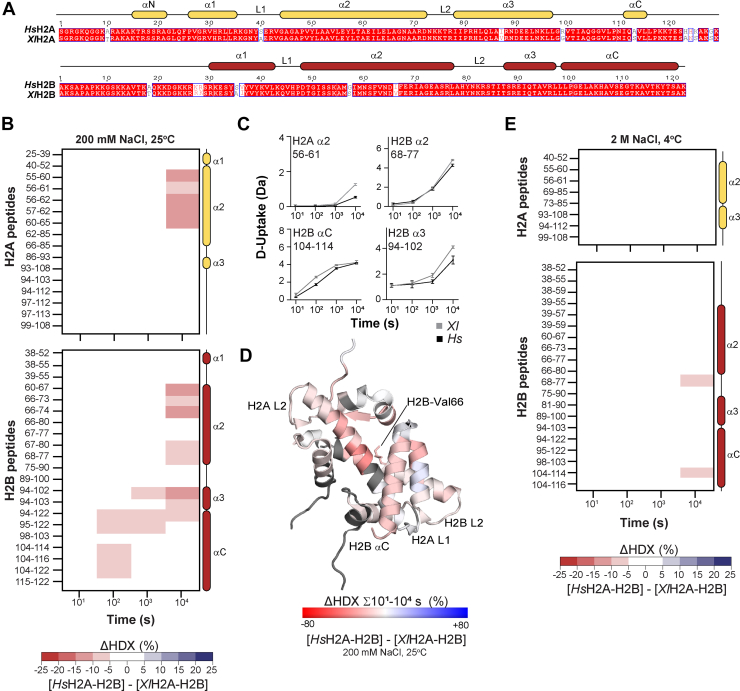
**Comparison of human and frog H2A-H2B dynamics.***A*, sequence alignment of *Hs* and *Xl*H2A-H2B with α-helices shown as *yellow* (H2A) or *red* (H2B) *cylinders*. Conserved residues (*shaded red*), similar residues (*red text*), and non-conserved substitutions (*black text*) are indicated. Alignment was made with Clustal Omega (60, 62, 63) and visualized with ESPript 3 (64). *B*, deuterium uptake difference (ΔHDX) between *Hs* and *Xl*H2A-H2B at 200 mM NaCl and 25 °C. Differences are ≥5% and have a *p*-value <0.01 in Welch’s *t* test (n = 3). *C*, example deuterium uptake (D-uptake) plots for H2A α2 and H2B α2, α3, and αC peptides. *Xl* is gray, and *Hs* is black. The y-axis is 80% of the maximum theoretical D-uptake. Error bars are ±2 SD with n = 3 or 4. *D*, *Hs*H2A-H2B from PDB 2CV5 showing ΔHDX (%) summed across all time points. Coloring is based on DynamX residue-level scripts; no statistical filters are applied. Residues without coverage are in *gray*. *E*, as for (B), but at 2 M NaCl and 4 °C.

**Fig. 3:**
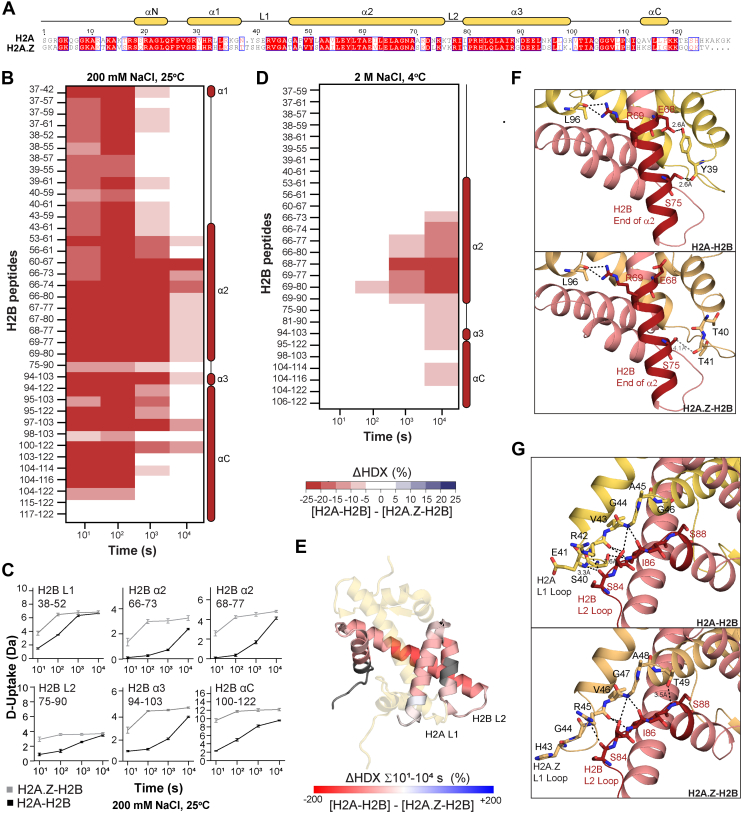
**Comparison of H2B dynamics when bound to core H2A or variant H2A.Z as a heterodimer in solution.***A*, sequence alignment of H2A and H2A.Z with α-helices shown as *yellow cylinders*. Conserved residues (*shaded red*), similar residues (*red text*), and non-conserved substitutions (*black text*) are indicated. Alignment was made with Clustal Omega (60, 62, 63) and visualized with ESPript 3 (64). *B*, deuterium uptake difference (ΔHDX) between H2B bound to H2A and H2A.Z at 200 mM NaCl and 25 °C. Differences are ≥5% and have a *p*-value <0.01 in Welch’s *t* test (n = 3). *C*, example deuterium uptake (D-uptake) plots for H2B L1, α2, L2, α3, and αC peptides at 200 mM NaCl and 25 °C. H2A.Z-H2B is *gray*, and H2A-H2B is *black*. The y-axis is 80% of the maximum theoretical D-uptake. Error bars are ±2 SD with n = 3 or 4. *D*, as for (*B*), but at 2 M NaCl and 4 °C. *E*, H2A-H2B from PDB 2CV5 showing ΔHDX (%) summed across all time points on H2B. Coloring is based on DynamX residue-level scripts; no statistical filters are applied. Residues without coverage are in *gray*. H2A is in transparent *yellow*. *F–G*, comparison of hydrogen bonds between H2A-H2B (*top*) (*yellow-salmon*) and H2A.Z-H2B (*bottom*) (*wheat-salmon*). Dimers were taken from nucleosome PDBs 2CV5 and 3AWA. (*F*) highlights hydrogen bonds for H2B residues 68 to 77 (*red*). *G*, highlights hydrogen bonds for H2B residues 78 to 90 (*red*). Key residues are shown as *sticks* and colored by atom, and hydrogen bonds are shown as *dotted lines*.

When performing HDX-MS, consistent chemical on-exchange and back-exchange rates for all samples are essential. The primary variables to control are time, pH, and temperature (22). Our workflow using histones with stable isotopes ensures that these variables are identical during the exchange reaction and during sample processing for histone H2A-H2B dimers, (H3-H4)_2_ tetramers, or octamers (24) (Table 1, Experiments A and B). We exchanged histone mixtures that were: ^15^N for core *Xl* histones, ^13^C for core *Hs* histones, and a wild-type isotopic distribution for variant *Hs* histone complexes. We also utilized common buffer stocks and parallel processing to standardize these variables across H2A-H2B, H3-H4, and histone octamer mixtures, enabling their comparison (Table 1, Experiment B). Our study also considered the impact of amino acid sequence, as our H2A-H2B homologs have substitutions, insertions, and deletions (Figs. 2*A* and 3*A*) (41). We compared the theoretical chemical on-exchange and back-exchange rates (calculated with HXPep (42)) of peptide homologs of the same length but with different amino acid sequences. If these rates differed for a peptide pair, we omitted them from the analysis. For *Hs* and *Xl* orthologs of all four core histones*,* we only had to omit peptides covering the C-terminal region of H2A that contains five substitutions (Fig. 2*A*). For *Hs*H2A and *Hs*H2A.Z; however, we had to omit all peptides as sequence differences altered the digestion pattern, leaving few peptide homologs, each with too many substitutions (Fig. 3*A*) (43, 44). This omission restricts our comparison of *Hs*H2A and *Hs*H2A.Z samples to other histone components (H2B and H3-H4), which are 100% identical between core and variant complexes.

**Table 1:** Overview of HDX-MS experiments

Experiment	**A**			**B**								
Mixture	1 – H2A-H2B			1 – H2A-H2B			2 – H3-H4			3 – Histone Octamer		
Proteins	*Xl* H2A-H2B	*Hs* H2A-H2B	*Hs* H2A.Z-H2B	*Xl* H2A-H2B	*Hs* H2A-H2B	*Hs* H2A.Z-H2B	*Xl* H3-H4	*Hs* H3-H4	*Hs* H3-H4	*Xl* H2A-H2B H3-H4	*Hs* H2A-H2B H3-H4	*Hs* H2A.Z-H2B H3-H4
Isotope	^15^N	^13^C	WT^a^	^15^N	^13^C	WT^a^	^15^N	^13^C	WT^a^	^15^N	^13^C	WT^a^
Exchange Conditions	200 mM NaCl, 25 °C			2 M NaCl, 4 °C								
	20 mM Tris-HCl, 1 mM EDTA, 1 mM DTT, pH 7.5											
	10^1^, 10^2^, 10^3^, 10^4^ s											
Significance Cutoff	≥5% D-uptake difference, *p*-value of <0.01 in a Welch’s t-test [n=3]											
No. of Peptides (Coverage %)												
H2A	16 (66.9)			8 (43.8)						8 (31.5)		
		*no peptide homologs*			*no peptide homologs*						*no peptide homologs*	
H2B	23 (67.5)			21 (67.5)						18 (61.1)		
		38 (66.4)			28 (66.4)						24 (66.4)	
H3							10 (40.0)			8 (52.6)		
											22 (69.6)	
H4							9 (47.1)			10 (68.6)		
											24 (89.2)	
Figure	2B and 3B			2E and 3D						5A		
	S2 and S4			S1, S3, and S5								

a WT stands for a natural isotopic abundance.

A summary of our HDX-MS experiments is in Table 1, and heat maps of “deuterium uptake” and “deuterium uptake difference” are shown in supplemental data (Supplemental Figs. S1–S5). In all cases, different heat maps have been filtered using a dual significance cutoff and only show differences ≥5% with a *p*-value <0.01 in Welch’s *t* test (n = 3 or 4) (45). In Experiment A, we compared H2A-H2B dimers at 200 mM NaCl and 25 °C, while in Experiment B, we compared H2A-H2B dimers and (H3-H4)_2_ tetramers to a histone octamer at 2 M NaCl and 4 °C. The dimer and tetramer histone complexes are described as “free” histones. We reconstituted the histone octamer and purified it by size-exclusion chromatography. We stabilized the octamer during the exchange reaction by using high protein concentration (5 μM octamer), low temperature (4 °C), and high ionic strength (2 M NaCl), in keeping with previous studies (6, 25). We used a relatively sizeable 10-fold dilution with a quench solution before injecting our samples into the mass spectrometer.

We can compare the exchange of histone octamers and free histones to determine whether the octamer remains stable during our exchange reaction. We did this for *Hs* core histones (Supplemental Fig. S1*A*), *Xl* core histones (Supplemental Fig. S1*B*), and *Hs* core histones with H2A replaced by H2A.Z (Supplemental Fig. S1*C*), with all samples at 2 M NaCl and 4 °C. In all cases, the histone octamer had less deuterium uptake than the corresponding free histones. The reduction was observed in all histones and was greater for H2A/H2A.Z-H2B than for H3-H4. Mapping the reduced deuterium uptake onto histone octamers extracted from nucleosome structures shows localization at known histone-histone interfaces, albeit not exclusively (Supplemental Fig. S1, *D*–*F*). These observations provide compelling evidence that the histone octamer exists in our exchange conditions and that high ionic strength mimics DNA to create a nucleosome-like conformation. While the kinetic stability of the H2A-H2B and H3-H4 interfaces in the salt-stabilized octamer is likely different from that in the DNA-bound octamer in the nucleosome, we can use high salt as a proxy for DNA in our experimental system.

### Conservative Substitutions in Orthologs Alter the Stability of the H2A-H2B Heterodimer

A direct comparison between H2A-H2B orthologs reveals small but significant differences in the deuteration of the conserved histone fold. At 200 mM NaCl and 25 °C, *Xl*H2A and H2B exhibited higher deuterium uptake than *Hs*H2A and H2B in both α2 helices, as well as in the nearby H2B α3 and αC helices (Fig. 2*B*, Supplemental Fig. S2). This increased deuterium uptake for *Xl*H2A-H2B compared to *Hs*H2A-H2B is clearly seen in representative plots for specific peptides (Fig. 2*C*). When mapped onto the H2A-H2B structure, these increases localize around the crossing of the two α2 helices in the H2A-H2B heterodimer (Fig. 2*D*). Given the high structural conservation between these two H2A-H2B orthologs, higher deuterium uptake at the interface between H2A and H2B suggests that the interface is “less stable” in the H2A-H2B heterodimer from frogs than from humans. The phrase “less stable” here and thereafter indicates that the protein has a conformational ensemble with enhanced solvent accessibility and/or reduced hydrogen-bond stability. Notably, there are no amino acid substitutions between orthologs in the α2 helix of H2A or the α3 and αC helices of H2B, but there are two in the α2 helix of H2B. There is a non-conservative serine-to-glycine substitution at residue 57 and a conservative valine-to-isoleucine substitution at residue 66 (Fig. 2*A*). It seems probable that one or both of these amino acid substitutions are the driver behind the differences observed between the orthologs. Given that in *Xl*H2A-H2B, serine-57 projects into solvent while valine-66 packs against hydrophobic residues in the α2 helix of H2A, such as leucine-58 (Fig. 2*D*), the valine-to-isoleucine substitution seems the likely candidate. Compared to isoleucine (human residue), valine (frog residue) lacks a methylene group, making it slightly less bulky and hydrophobic. The substitution of valine with isoleucine in histones, as they evolved from frogs to humans, seemingly resulted in a more stable H2A-H2B heterodimer. The known folding-upon-binding mechanism reported for the H2A-H2B may explain how changing a single amino acid can have far-reaching effects within the heterodimer.

We can similarly compare H2A-H2B and H3-H4 orthologs as free histones or histones reconstituted into the octamer. These were exchanged at 2 M NaCl and 4 °C, where free H2A-H2B remains a heterodimer, and two H3-H4 come together to form a (H3-H4)_2_ tetramer (25). We observed almost identical deuterium uptake between the *Xl* and *Hs* histone complexes. Data for H2A-H2B heterodimer, (H3-H4)_2_ tetramer, and the octamer are shown in Figure 2*E* and Supplemental Figs. S3. The relatively lower stability of *Xl*H2A-H2B observed at 200 mM NaCl and 25 °C was no longer observed at 2 M NaCl and 4 °C, in either free histones or the histone octamer. Two primary factors likely contribute to this result: the lowered temperature reduces the chemical on-exchange rate (20, 46), and the higher ionic strength likely stabilizes the histone structure [as shown previously for yeast H2A-H2B (47)]. These factors mask the differences between the frog and human H2A-H2B heterodimer.

### Replacing H2A with H2A.Z Drastically Enhances H2B Dynamics

The slight difference in the deuteration of frog and human H2A-H2B orthologs likely reflects the few residue substitutions between them. We can further test this paradigm by comparing *Hs*H2B bound to *Hs*H2A or its paralogous variant *Hs*H2A.Z. Human H2A and H2A.Z are less similar, with only 60% sequence similarity and a notable single-residue insertion in the H2A.Z L1 DNA-binding loop (Fig. 3*A*). A direct comparison of H2A-H2B and H2A.Z-H2B reveals a widespread increase in deuterium uptake for H2B bound to the variant. At 200 mM NaCl and 25 °C, H2B bound to H2A.Z had higher deuterium uptake than when bound to H2A in almost all peptides recovered in our experiment (Figs. 3*B*, Supplemental Fig. S4). The significant peptides redundantly cover the end of α1 through to αC, and increased deuterium uptake was observed at all exchange time points. Representative plots for specific peptides in the same experiment show that the absolute magnitude of the increase (Fig. 3*C*) is far greater than that between frog and human H2B. Mapping the increase onto the H2A-H2B structure reinforces that it is not localized and occurs throughout H2B (Fig. 3*E*). The higher deuterium uptake for H2B bound to H2A.Z compared to H2A suggests that the variant heterodimer is less stable than its canonical counterpart. While this has repercussions for variant function, it also supports the idea that the number of residue substitutions may correlate with differences among structurally similar H2A-H2B homologs.

**Fig. 4:**
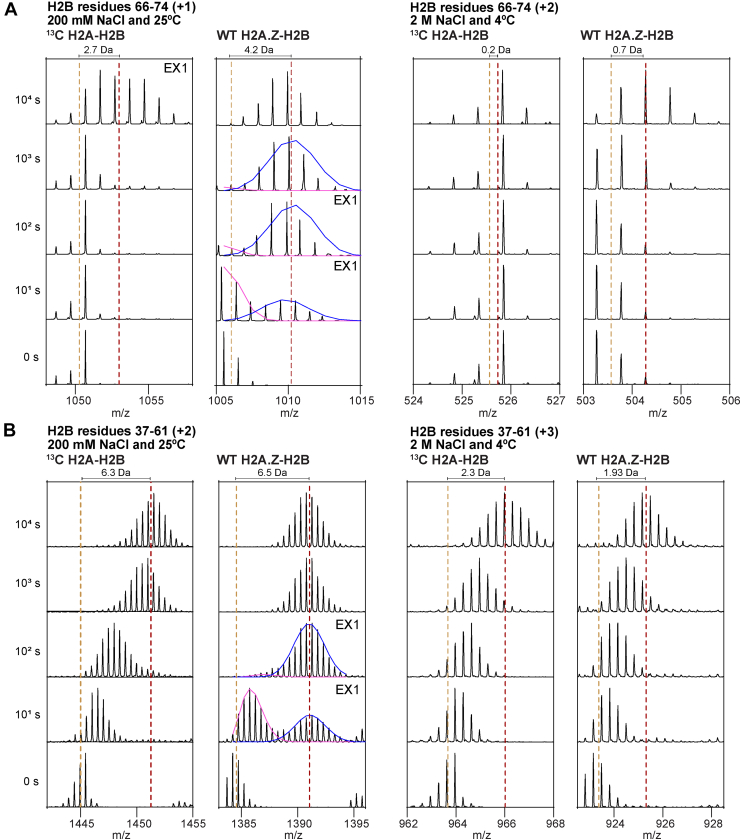
**Example peptide spectra for H2B from either a H2A-H2B or H2A.Z-H2B heterodimer.***A*, spectra for H2B peptide covering residues 66 to 74 at 200 mM NaCl and 25 °C (*left*), and 2 M NaCl and 4 °C (*right*). *B*, spectra for H2B peptide covering residues 37 to 61 at 200 mM NaCl and 25 °C (*left*), and 2 M NaCl and 4 °C (*right*). EX1 spectra are labeled and, where possible, fit with HXExpress (65). *Yellow dotted lines* mark the centroid of non-deuterated controls. *Red dotted lines* mark the centroid after 10^4^ s of deuteration. As peptides at different NaCl concentrations had different charged states, the Dalton mass shift is labeled.

The enhanced deuteration of H2A.Z-H2B compared to H2A-H2B is due to a higher frequency of cooperative unfolding events. These events cause groups of backbone amides to exhibit correlated exchange, resulting in either none or all of them being deuterated. This exchange manifests as EX1 kinetics, characterized by multimodal peptide spectra (48). We have previously reported EX1 kinetics for H2A-H2B and demonstrated that they are due to local unfolding rather than complete heterodimer dissociation (47). When we replaced H2A with H2A.Z, we observed EX1 kinetics at earlier time points and in more H2B peptides (Fig. 4). In canonical H2A-H2B, EX1 was detected only for H2B residues 66 to 74, while for variant H2A.Z-H2B, it was detected throughout the entire H2B sequence. Spectra for the H2B peptide residues 66 to 74 illustrate how EX1 occurs much earlier for H2A.Z-H2B than for H2A-H2B (Fig. 4*A* *left*). The bimodal spectra for H2A-H2B are challenging to fit due to the presence of the ^13^C label (24). Spectra for the H2B peptide 37 to 61 show that EX1 occurs in additional regions of H2A.Z-H2B compared to H2A-H2B (Fig. 4*B* *left*).

The dynamic difference between core H2A-H2B and variant H2A.Z-H2B as heterodimers was also observed when the exchange was performed at 2 M NaCl and 4 °C (Figs. 3*D*, Supplemental Fig. S5*A*). Compared to exchange at 200 mM NaCl and 25 °C, however, the number of peptides with a significant H2A.Z-induced increase in deuterium uptake was less, and the differences were seen at later time points (compare Fig. 3, *B* and *D*). This results from the lower chemical on-exchange rate (20, 46) and/or salt-mediated stabilization of histone structure (47). Consistent with the latter, we observed no bimodal spectra for H2B under high-salt conditions (Fig. 4, *A* and *B right*). The region of H2B that maintained enhanced deuteration in the variant heterodimer at high salt covers the end of α2 and L2, primarily residues 69 to 77 and, to a lesser extent, residues 78 to 90 (Fig. 3*G*). Notably, these regions of H2B have fewer hydrogen bonds with H2A.Z than H2A when looking at heterodimer structures taken from their respective nucleosomes (Fig. 3, *E* and *F*). The replacement of H2A Y39 with H2A.Z T40 and T41 removes two hydrogen bonds with H2B E68 and S75 (Fig. 3*F*). The H2B L2 loop also has fewer hydrogen bonds at the S84 end but an additional hydrogen bond for S88 when comparing H2A.Z-H2B and H2A-H2B (Fig. 3*G*). The deuteration differences that are large enough to persist at high salt concentrations thus overlap with regions of H2B that show distinct hydrogen bonding with H2A and H2A.Z in the nucleosome. Overall, this suggests that these hydrogen-bonding differences between H2A-H2B and H2A.Z-H2B also occur in the heterodimer state. The large magnitude of the difference in deuteration in these H2B regions contrasts with the relatively small differences between human and frog H2A-H2B orthologs.

**Fig. 5:**
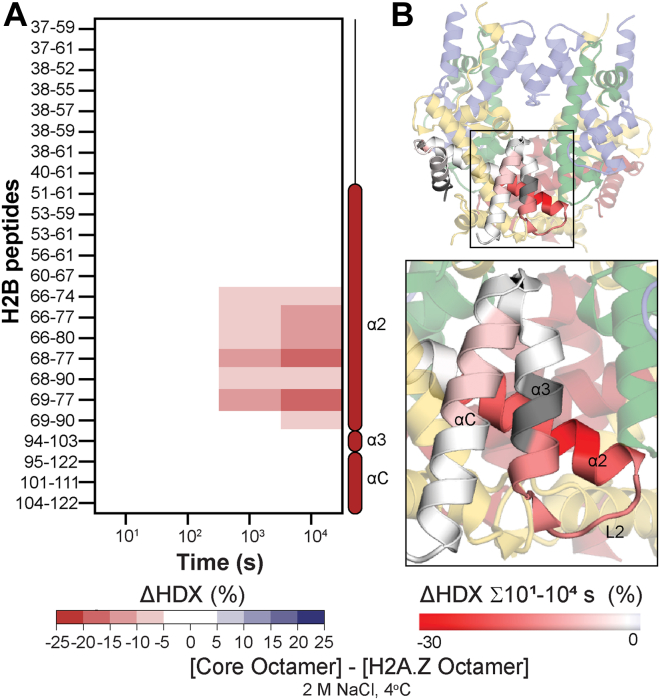
**Comparison of H2B dynamics in the histone octamer with core H2A or variant H2A.Z at 2 M NaCl and 4 °C.***A*, deuterium uptake difference (ΔHDX) between H2B in the histone octamer with core H2A or variant H2A.Z, at 2 M NaCl and 4 °C. Differences are ≥5% and have a *p*-value <0.01 in Welch’s *t* test (n = 3). *B*, histone octamer from PDB 2CV5. H2A is *yellow*, H3 is *blue*, and H4 is *green*. H2B coloring shows ΔHDX (%) summed across all time points. H2B coloring is based on DynamX residue-level scripts; no statistical filters are applied. Residues without coverage are in *gray*. Zoom-in shows H2B α2, L2, α3, and αC.

To ascertain whether the enhanced dynamics of H2A.Z-H2B are further maintained in a nucleosome-like environment, we can compare histone octamers with H2A to those with H2A.Z (Fig. 5, Supplemental Fig. S5*B*). In this experiment, both copies of H2A-H2B were replaced with H2A.Z-H2B. We again observed an increase in deuterium uptake for H2B in the variant octamer compared to the core octamer for peptides covering the end of α2 and L2 (Fig. 5*A*). This region is similar to that seen for the heterodimer (compare Figs. 3*E* and 5*A*). In contrast, the deuterium uptake of H3-H4 was identical between the variant and core octamers (Supplemental Fig. S5*B*). This result suggests that the variant and core octamers have similar interactions between H2A/H2A.Z-H2B and H3-H4 and, thus, similar octamer stabilities. The region of H2B that has an H2A.Z-induced increase in deuteration is somewhat exposed in the octamer and is involved in DNA binding in the nucleosome (Fig. 5*B*).

### Replacing H2A with H2A.Z Enhances Nucleosome Dynamics

**Fig. 6:**
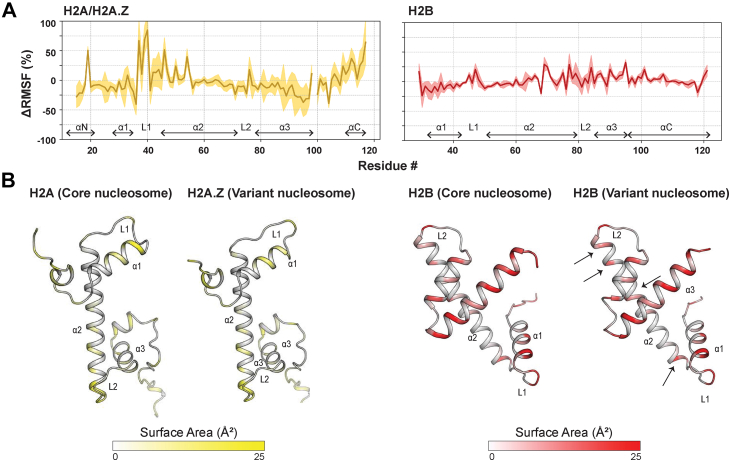
**MD comparison of core and variant-containing nucleosomes.***A*, comparison of residue flexibility between H2A-H2B and H2A.Z-H2B in the nucleosome. ΔRMSF = [RMSF(H2A.Z-H2B) - RMSF(H2A-H2B)]/RMSF(H2A-H2B) × 100, where positive values indicate increased flexibility in the variant compared to the core, and vice versa. ΔRMSF was calculated for H2A residues 13 to 118 and H2B residues 29 to 121. The *solid lines* show the average ΔRMSF over six trials, and the *shaded lines* show ±SD. *B*, comparison of solvent-accessible surface area of H2A/H2A.Z and H2B in the nucleosome. The intensity of the *yellow/red* color indicates higher exposure to solvent. *Arrow marks* indicate the residues with increased exposed surface area in H2A.Z-H2B nucleosomes compared to H2A-H2B nucleosomes. Actual values reported in Supplemental Tables S2 and S3.

To extend our comparison of H2A-H2B and H2A.Z-H2B, we performed MD simulations comparing core and H2A.Z-containing variant nucleosomes. We constructed two model systems: core human nucleosomes and double-variant human nucleosomes, where both H2A.Z-H2B are replaced by H2A-H2B. Each system was simulated for 450 ns in triplicate (containing two copies of each dimer) at pH 7.5 and 200 mM NaCl. RMSD was used to assess the nucleosome's overall structural stability and compactness. The results indicated that all simulations reached equilibrium and remained stable throughout the simulation (Supplemental Fig. S6*A*). To evaluate residue-level flexibility, we calculated and compared the RMSF of homologous residues between H2A and H2A.Z (as aligned in Fig. 3*A*) and H2B (all residues identical) (Fig. 6*A*, Supplemental Fig. S6*B*). This comparison revealed that H2A.Z had increased flexibility compared to H2A in the L1 and α2 regions (Fig. 6*A* *left*). H2B bound to H2A.Z in the variant nucleosome also exhibited higher flexibility, particularly in L1, α2, L2, and α3 regions, compared to H2B bound to H2A in the core nucleosome (Fig. 6*A*, *right*). The increased flexibility of nucleosomal H2A.Z-H2B compared to nucleosomal H2A-H2B is consistent with previously reported MD studies (49, 50, 51). B-factor calculations, derived from the RMSF of each residue, can also be compared across the structures of H2A, H2A.Z, and H2B (Supplemental Fig. S6*C*). The enhanced flexibility of H2A.Z-H2B in the variant nucleosome compared to the core nucleosome is qualitatively similar to our HDX-MS data with histone octamers. Overall, the MD suggests that H2A.Z-H2B is more dynamic than H2A-H2B in the nucleosome.

We further assessed the exposure of nucleosomal H2A-H2B or H2A.Z-H2B by calculating the solvent-accessible surface area (Fig. 6*B* and Supplemental Tables S2 and S3). We observed that the L1 of H2A.Z in the variant nucleosome had increased surface area compared to H2A in the core nucleosome (Fig. 6*B* *left*). At the same time, the nearby α2 and L2 regions of H2B bound to H2A.Z in the variant nucleosome had increased surface area compared to H2B bound to H2A in the core nucleosome (Fig. 6*B* *right*). Increased solvent-accessible surface area may be attributed to fewer contacts between H2A/H2A.Z and H2B (as observed in the respective nucleosome crystal structures, Fig. 5, *F* and *G*), and/or reduced contacts with DNA. To assess the former, we calculated all hydrogen bonds and residue-residue contacts for H2A/H2A.Z and H2B in core or variant nucleosomes (Fig. S7). We observed fewer hydrogen bonds for the variant nucleosome compared to the core nucleosome, suggesting a weaker subunit interaction for H2A.Z and H2B than for H2A and H2B (Supplemental Fig. S7*A*). Although the residue-residue contact maps were very similar, one region did show a more dispersed pattern of interaction in the variant nucleosome compared to the core nucleosome (Supplemental Fig. S7*B*). This region was the L1 loop of H2A/H2A.Z and the α2–L2 region of H2B. Notably, this is the same region of H2B that we observed increased deuteration with H2A.Z than with H2A in the dimer and octamer at 2 M NaCl (Figs. 3*D* and 5*A*).

**Fig. 7:**
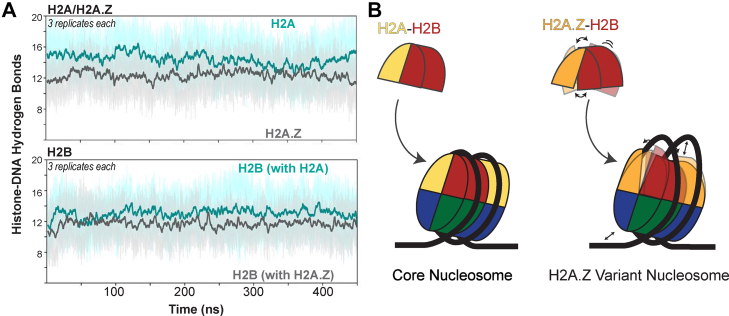
**MD comparison of H2A-H2B and H2A.Z-H2B hydrogen bonding with DNA in the nucleosome.***A*, Hydrogen bond plot comparing the number of polar interactions between H2A/H2A.Z-H2B and DNA in the nucleosome. The *teal* and *gray solid lines* show the average number of hydrogen bonds over six trials for the core and variant nucleosome, respectively. The *shaded lines* show ±SD. *B*, H2A/H2A.Z-H2B schematic showing that increased dynamics in the variant dimer persist in the histone octamer and nucleosome and influence DNA interactions. Histones are *yellow, orange, red, blue,* and *green* for H2A, H2A.Z, H2B, H3, and H4, respectively. DNA is *black*.

Further, to more explicitly quantify nucleosomal DNA interactions, we calculated the number of hydrogen bonds formed between both copies of H2A/H2A.Z-H2B and DNA. The hydrogen bond analysis revealed a significant reduction in the polar interactions formed between histones and DNA for the variant nucleosome (Fig. 7*A*). The number of hydrogen bonds between H2A.Z or H2B and DNA in the variant nucleosomes is reduced by an average of five and two interactions, respectively, compared to those between H2A or H2B and DNA in the core nucleosomes. This reduction in histone-DNA interactions supports a model in which the enhanced dynamics of the H2A.Z-H2B dimer relative to the H2A-H2B dimer manifest in the nucleosome as compromised DNA interactions (Fig. 7*B*). This ultimately makes DNA more accessible in the variant nucleosome than in the core nucleosome. Despite high structural homology, the vast sequence differences between H2A and H2A.Z alter dynamics that affect the histone heterodimer, the histone octamer, and the nucleosome.

## Discussion

Many prior studies have investigated the physical effects of replacing core H2A with variant H2A.Z in the nucleosome (49, 50, 51, 52, 53, 54). We have extended these studies and provide compelling evidence from HDX-MS and MD that variant substitution effects originate in the histone heterodimer and persist, to some degree, in the histone octamer and the nucleosome. We considered different oligomeric states as the isolated H2A-H2B heterodimer adopts a distinct conformation compared to its structured integration within the nucleosome, highlighting its dynamic and flexible nature outside the nucleosome context (55). We have demonstrated that replacing H2A with H2A.Z in the H2A-H2B heterodimer significantly reduces the stability of hydrogen bonds formed by amides in the H2B backbone (Fig. 3). H2B in the variant dimer more extensively and more frequently unfolds than H2B in the canonical dimer (Fig. 4). Prior circular dichroism studies are consistent in showing that at moderate ionic strength, H2A.Z-H2B does not achieve a stable fold while H2A-H2B does (56). Our results highlight how protein dynamics are drastically influenced by binding interactions rather than being purely an intrinsic property of protein sequence. The influence of a binding partner (H2A or H2A.Z in this case) may be particularly pronounced for complexes like histone heterodimers, where protein folding is closely tied to binding, and interfaces are proportionally large.

Our work also recapitulates previous findings that histone dynamics are modulated by ionic strength (47). This NaCl-mediated stabilization is frequently used *in vitro* to drive the specificity of histone interactions with either DNA or other proteins (Fig. 1*A*) (6, 26). Such stabilization is likely achieved in the cell through interactions with histone chaperones. Histone chaperones typically bind histones with tight affinity and often contain disordered acidic regions that contribute to histone stabilization and act as a DNA mimic (47, 57, 58). For example, histone chaperones Nap1 and Chz1 have been shown to stabilize the H2A-H2B and H2A.Z-H2B heterodimers, respectively (17, 47). Increasing the ionic strength eliminated the subtle difference between frog and human H2A-H2B, but not the more considerable difference between core H2A-H2B and variant H2A.Z-H2B (Figs. 2 and 3). H2B in the variant dimer remained more dynamic than the core dimer in one specific region, the H2B α2 and L2 loop. This result is remarkably mirrored by NMR data showing that the H2B L2 loop is unstable in H2A.Z-H2B even when bound to the Chz1 chaperone (59). The enhanced instability of the H2B L2 loop in the variant dimer thus persists in the presence of high ionic strength or chaperone binding.

We further demonstrate that the relative instability of the H2B L2 loop is also seen in a histone octamer that is stabilized by low temperature and high ionic strength (Fig. 5). A comparison of free H2A-H2B dimers or (H3-H4)_2_ tetramers with the histone octamer showed that these conditions serve as an appropriate surrogate for the nucleosome (Supplemental Fig. S1). The high NaCl concentration has a similar effect to DNA in stabilizing interactions between H2A-H2B and H3-H4. Related to this, an essential aspect of our results is that H2A.Z incorporation into the histone octamer does not alter the (H3-H4)_2_ tetramer (Supplemental Fig. S5*B*). This result suggests that H2A.Z does not affect the interaction between H2A-H2B and H3-H4, explaining why H2A.Z does not significantly alter chromatin organization and nucleosome assembly (Fig. 1). The importance of local differences between H2A-H2B and H2A.Z-H2B in the nucleosome however is underscored by work with the Swc2 chaperone, which performs unidirectional exchange of H2A-H2B for H2A.Z-H2B within the nucleosome (54, 61). It is intriguing to note that recognition of H2A-H2B nucleosomes as a substrate (but not H2A.Z-H2B nucleosomes) relies on residues in the precise region that we showed had altered deuterium uptake between core and variant heterodimers and octamers at 2 M NaCl.

Our MD simulations comparing core and variant nucleosomes further reinforce the idea that H2A.Z locally enhances dynamics around the H2B α2 and L2 loop. Prior MD studies have yielded complementary results. Bowerman and Wereszczynski (2016) reported that the increase in nucleosome dynamics mediated by H2A.Z arises primarily through the H2A.Z L1 loop, which significantly alters the allosteric communication with the DNA (49). Kniazeva *et al.* (2022) reported that H2A.Z enhances the conformational plasticity of the H2A.Z-H2B dimer relative to the canonical H2A-H2B pair (50). Li *et al.* (2023) reported that the tails of H2A.Z play critical roles in DNA unwrapping and nucleosome gapping (51). In our MD simulation, we calculated solvent-accessible surface area and analyzed contacts between H2A/H2A.Z and H2B, as well as those between H2A/H2A.Z-H2B and DNA (Figs. 6 and 7, and Supplemental Fig. S7). We found that the variant had fewer hydrogen bonds than its canonical counterpart in both cases. Reduced contacts between H2A.Z and H2B, and reduced DNA binding by H2A.Z-H2B may explain how H2A.Z promotes chromatin accessibility for remodeling and transcription.

Identifying the H2A.Z L1 loop as a site with altered dynamics is perhaps not surprising given its low conservation with H2A (Fig. 3*A*). The loop contains several residue substitutions and a single-residue insertion for H2A.Z. These differences compromise the interactions between the L1 loop of H2A.Z and the L2 loop of H2B, ultimately limiting the stability of their interaction with DNA. We observed this in our sequential nucleosome assembly assay, where H2A.Z-H2B has fewer histone-DNA by-products than H2A-H2B, suggesting a weaker interaction with DNA (Fig. 1, *C* and *D*). Further, the extra residue in the H2A.Z L1 loop also supports a variant-specific interaction between the two copies of H2A.Z in a double-variant nucleosome (61). It is reasonable to assume that the enhanced dynamics of the H2A.Z L1 and H2B L2 loops would facilitate this interaction that promotes variant nucleosome stability. The interaction may partially counteract the destabilizing effect of reduced interactions between H2A.Z-H2B and DNA.

Our comparative analysis reveals that proteins with high structural and sequence conservation can exhibit vastly different dynamics, likely contributing to their unique functions. We have demonstrated that replacing canonical H2A-H2B with variant H2A.Z-H2B in various chromatin complexes results in enhanced dynamics that alter protein-protein and protein-DNA interactions. Our results improve our understanding of how histone variants influence nucleosome architecture and stability, ultimately affecting chromatin accessibility within the cell.

## Data Availability

HDX data described in the manuscript are in Supplemental Table S1, and all raw files have been deposited into ProteomeXchange via the PRIDE partner repository (PXD062956). All data will be shared upon request to Sheena D’Arcy at the University of Texas at Dallas (sheena.darcy@utdallas.edu).

## Supplemental Data

This article contains supplemental data.

## Conflict of Interest

The authors declare that they do not have any conflicts of interest with the content of this article.
