# Supplementary material for: Histone Variant H2A.Z Enhances Histone and Nucleosome Dynamics
Source: Mol Cell Proteomics. 2026 Jan 29;25(3):101518. doi: 10.1016/j.mcpro.2026.101518 (PMC12969112; doi:10.1016/j.mcpro.2026.101518)
Supplement: Supplemental Data [file mmc1.docx]

**Supplemental Information**

**Title:** Histone variant H2A.Z enhances histone and nucleosome dynamics

**Authors:** Juliana Kikumoto Dias^*^, Prabavi Shayana Dias^*^, Rakhat Alakenova^#^, Charles Mariasoosai^#^, Claudia Claridy, Sameeha Gazi, Hedieh Torabifard, Sheena D'Arcy^**^

**Affiliations:**

Department of Chemistry & Biochemistry, The University of Texas at Dallas, Richardson, TX, 75080, USA

^*^ or ^#^ equal contribution

^**^ corresponding author, sheena.darcy@utdallas.edu

**Running Title:** Effect of H2A.Z on dynamics of histone complexes

**Table S1:** **Summary and state data for HDX-MS experiments** (attached Excel file).

**Table** **S2:** **Solvent-accessible surface area (Å^2^) for each residue of H2B in core and variant nucleosomes.** The first column lists the H2B residue, the second and third columns the solvent-accessible surface area for H2A-bound and H2A.Z-bound H2B in the nucleosome, respectively, and the fourth column the difference between them. All values are averaged over six trials.

| **Residue** | **H2B in core nucleosome (Å^2^)** | **H2B in variant nucleosome (Å^2^)** | **Difference**  **(variant – core) (Å^2^)** |
| --- | --- | --- | --- |
| SER29 | 4.81 | 12.43 | 7.62 |
| LYS31 | 12.02 | 14.98 | 2.96 |
| GLU32 | 4.97 | 6.02 | 1.05 |
| SER33 | 2.82 | 3.46 | 0.64 |
| TYR34 | 0.61 | 1.00 | 0.39 |
| SER35 | 3.01 | 3.13 | 0.13 |
| VAL36 | 5.09 | 5.24 | 0.15 |
| LEU42 | 0.09 | 0.11 | 0.02 |
| LYS43 | 10.11 | 10.46 | 0.35 |
| VAL45 | 4.27 | 4.41 | 0.15 |
| GLY50 | 0.86 | 0.97 | 0.11 |
| ILE51 | 0.36 | 0.49 | 0.13 |
| SER52 | 1.17 | 1.25 | 0.09 |
| LYS54 | 9.81 | 13.75 | 3.94 |
| MET56 | 2.51 | 2.53 | 0.02 |
| GLY57 | 2.03 | 2.30 | 0.27 |
| ILE58 | 0.18 | 0.28 | 0.10 |
| MET59 | 0.08 | 0.09 | 0.01 |
| ASN60 | 1.44 | 1.88 | 0.44 |
| PHE62 | 0.30 | 0.32 | 0.02 |
| VAL63 | 0.06 | 0.11 | 0.05 |
| ASN64 | 4.25 | 4.71 | 0.46 |
| ASP65 | 1.20 | 1.70 | 0.50 |
| PHE67 | 0.82 | 1.29 | 0.47 |
| GLU68 | 3.32 | 5.75 | 2.43 |
| ARG69 | 6.88 | 7.81 | 0.93 |
| ILE70 | 0.04 | 0.19 | 0.15 |
| ALA71 | 0.01 | 0.23 | 0.23 |
| ALA74 | 0.00 | 0.04 | 0.04 |
| SER75 | 1.09 | 1.57 | 0.48 |
| ARG76 | 6.47 | 8.29 | 1.82 |
| ALA78 | 0.06 | 0.21 | 0.15 |
| HIS79 | 4.52 | 7.06 | 2.54 |
| TYR80 | 4.04 | 4.29 | 0.26 |
| ASN81 | 3.43 | 4.14 | 0.71 |
| LYS82 | 16.96 | 19.82 | 2.86 |
| ARG83 | 3.43 | 3.47 | 0.03 |
| SER84 | 2.14 | 3.07 | 0.93 |
| THR85 | 0.73 | 1.10 | 0.37 |
| ILE86 | 0.03 | 0.27 | 0.25 |
| THR87 | 3.20 | 3.60 | 0.40 |
| ARG89 | 8.04 | 8.21 | 0.17 |
| ILE91 | 0.01 | 0.03 | 0.03 |
| VAL95 | 0.00 | 0.01 | 0.01 |
| LEU98 | 2.53 | 2.75 | 0.22 |
| PRO100 | 2.28 | 3.23 | 0.95 |
| ALA104 | 0.52 | 0.61 | 0.09 |
| LYS105 | 14.75 | 15.10 | 0.35 |
| VAL108 | 2.93 | 3.00 | 0.08 |
| SER109 | 5.53 | 5.57 | 0.04 |
| THR112 | 6.71 | 6.82 | 0.11 |
| LYS113 | 11.75 | 11.84 | 0.09 |
| ALA114 | 0.32 | 0.35 | 0.03 |
| LYS117 | 11.89 | 12.01 | 0.12 |
| SER120 | 8.79 | 8.99 | 0.21 |
| ALA121 | 9.65 | 13.61 | 3.96 |

**Table** **S3:** **Solvent-accessible surface area (Å^2^) for each residue of H2A/H2A.Z in core and variant nucleosomes.** The first and third columns list the H2A and H2A.Z residues, respectively. The second and fourth columns list the solvent-accessible surface areas for H2A and H2A.Z in the nucleosome, respectively. The fifth column lists the difference between them. All values are averaged over six trials.

| **H2A Residue** | **H2A in core nucleosome (Å^2^)** | **H2A.Z Residue** | **H2A.Z in variant**  **nucleosome (Å^2^)** | **Difference**  **(variant – core) (Å^2^)** |
| --- | --- | --- | --- | --- |
| LYS13 | 8.85 | LYS15 | 10.19 | 1.34 |
| ALA14 | 0.99 | ALA16 | 1.78 | 0.79 |
| SER19 | 6.27 | GLN21 | 11.82 | 5.55 |
| ARG20 | 6.66 | ARG22 | 8.39 | 1.73 |
| ARG32 | 5.78 | ARG34 | 8.13 | 2.35 |
| LEU33 | 3.88 | HIS35 | 4.66 | 0.79 |
| LEU34 | 0.05 | LEU36 | 0.26 | 0.21 |
| GLY37 | 0.52 | ARG39 | 5.65 | 5.13 |
| - | - | THR41 | 1.16 | - |
| TYR39 | 1.98 | SER42 | 3.05 | 1.08 |
| SER40 | 0.82 | HIS43 | 10.02 | 9.20 |
| ARG42 | 1.81 | ARG45 | 2.16 | 0.35 |
| GLY46 | 1.51 | THR49 | 3.28 | 1.77 |
| ALA47 | 0.01 | ALA50 | 0.05 | 0.04 |
| TYR50 | 0.76 | TYR53 | 0.89 | 0.13 |
| LEU51 | 0.00 | SER54 | 0.12 | 0.12 |
| VAL54 | 0.00 | ILE57 | 0.02 | 0.02 |
| LEU55 | 0.05 | LEU58 | 0.12 | 0.07 |
| THR59 | 0.01 | THR62 | 0.02 | 0.01 |
| LEU65 | 4.95 | LEU68 | 6.19 | 1.25 |
| ASN68 | 7.29 | ASN71 | 7.49 | 0.20 |
| ALA69 | 1.13 | ALA72 | 1.57 | 0.45 |
| ASP72 | 11.70 | ASP75 | 11.78 | 0.08 |
| ASN73 | 7.61 | LEU76 | 8.75 | 1.15 |
| THR76 | 5.61 | LYS79 | 10.51 | 4.90 |
| PRO80 | 0.65 | PRO83 | 1.22 | 0.58 |
| HIS82 | 0.76 | HIS85 | 0.83 | 0.08 |
| GLN84 | 0.99 | GLN87 | 1.24 | 0.25 |
| ALA86 | 0.15 | ALA89 | 0.40 | 0.25 |
| GLU91 | 9.42 | GLU94 | 10.48 | 1.06 |
| ASN94 | 2.66 | ASP97 | 3.62 | 0.96 |
| GLU98 | 2.77 | LYS101 | 15.52 | 12.75 |
| ARG99 | 11.04 | - | - | - |
| THR101 | 1.58 | THR104 | 3.30 | 1.72 |
| ILE102 | 0.53 | ILE105 | 0.87 | 0.35 |
| ALA103 | 0.48 | ALA106 | 1.02 | 0.54 |
| GLY105 | 0.02 | GLY108 | 0.07 | 0.05 |
| GLY106 | 2.23 | GLY109 | 2.57 | 0.34 |
| LEU108 | 4.91 | ILE111 | 6.22 | 1.31 |
| ASN110 | 8.17 | HIS113 | 9.19 | 1.02 |
| ILE111 | 4.25 | ILE114 | 4.37 | 0.12 |
| ALA113 | 8.26 | LYS116 | 19.37 | 11.11 |
| LEU115 | 0.24 | LEU118 | 0.34 | 0.10 |
| LEU116 | 8.07 | ILE119 | 9.00 | 0.92 |


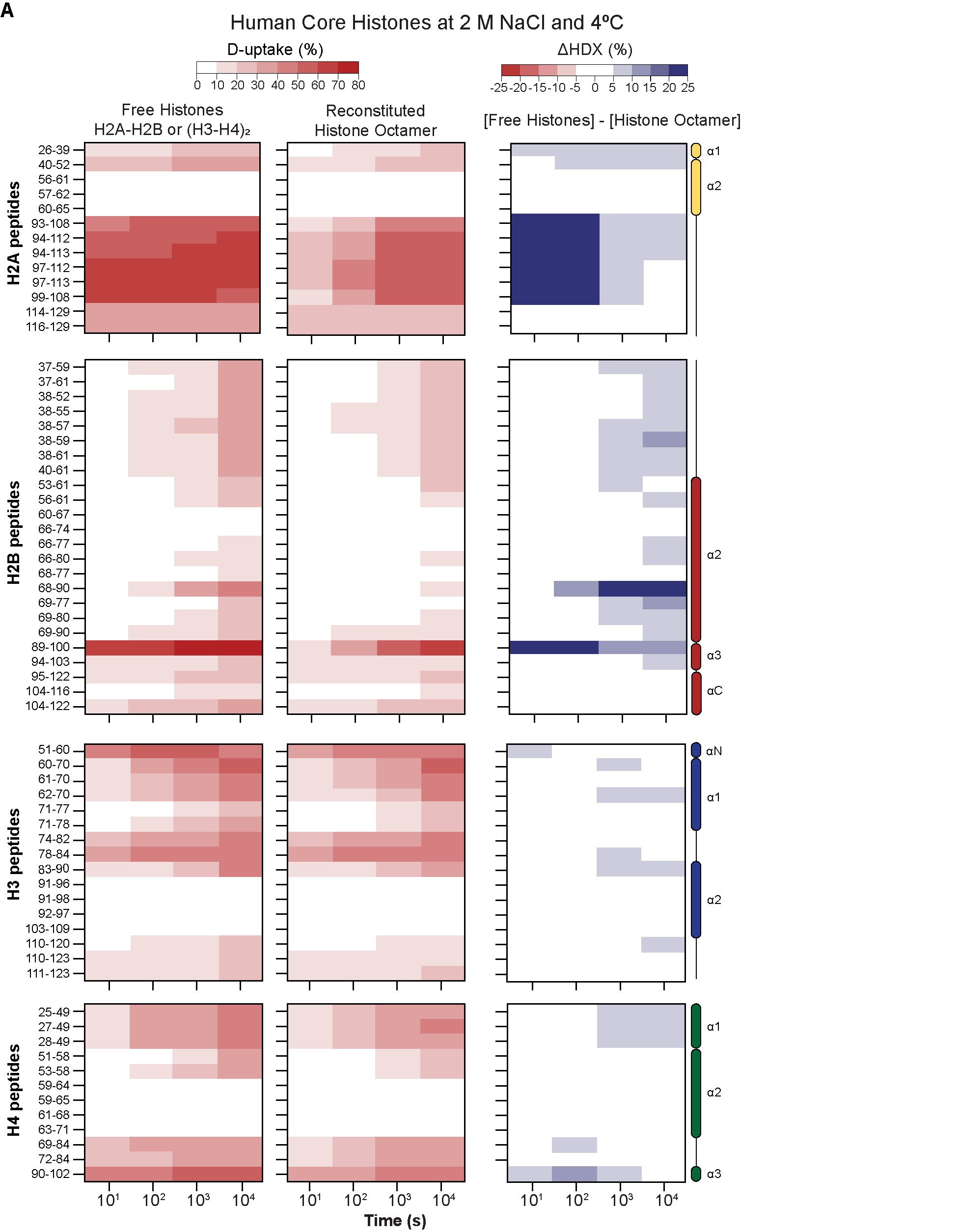

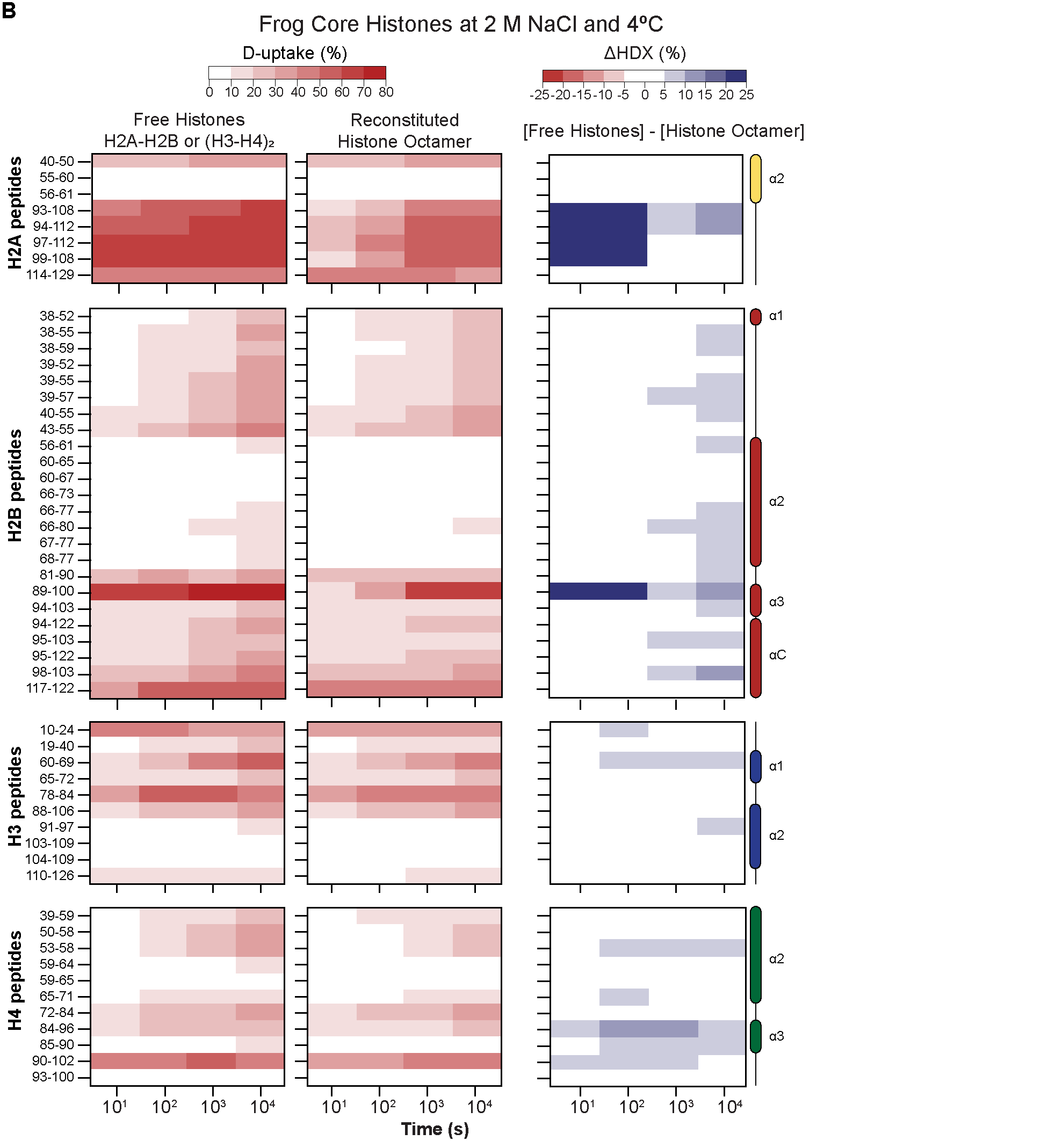

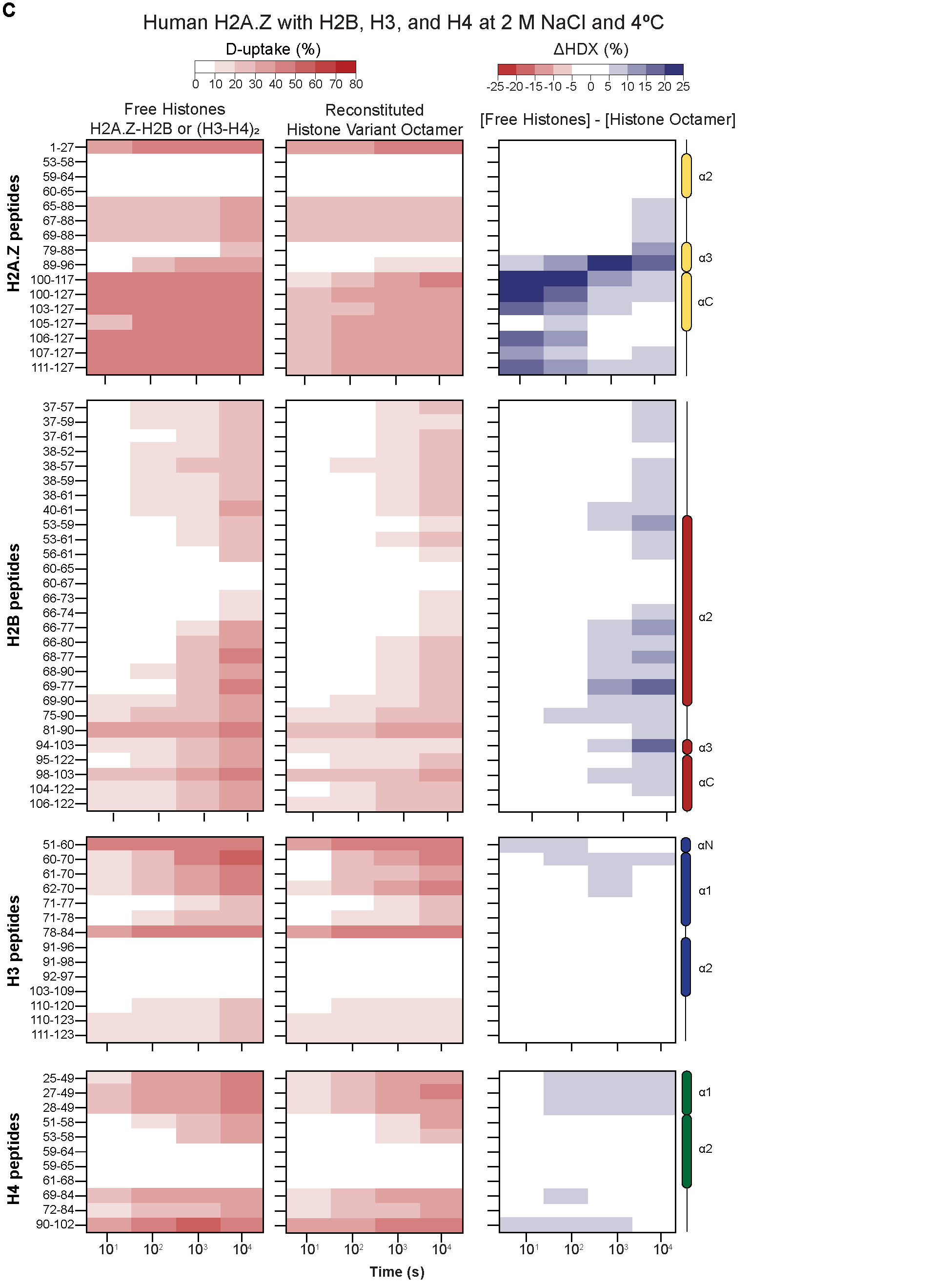

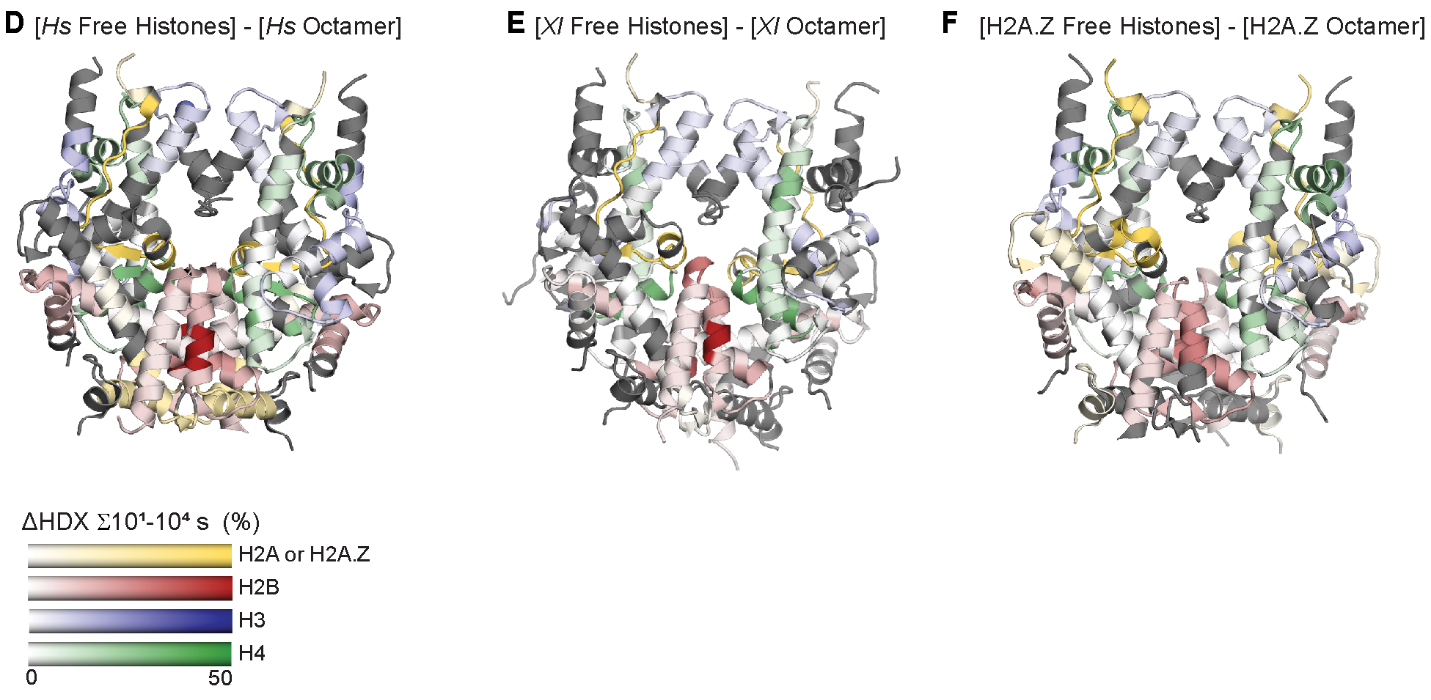


**Figure S1: Dynamic changes upon octamer formation for *Hs* core octamer (A), *Xl* core octamer (B) and H2A.Z variant octamer (C) at 2 M NaCl and 4^o^C. (A-C)** *Left* panel shows deuterium uptake (%) for free histones. *Middle* panel shows deuterium uptake (%) for the reconstituted histone octamer. *Right* panel shows the deuterium uptake difference (ΔHDX) between the free histones and the histone octamer. Differences are ≥5% and have a *p*-value <0.01 in Welch’s t-test (n=3). Histone α-helices are shown as yellow (H2A), red (H2B), blue (H3), or green (H4) cylinders. **(D-F)** Histone octamers showing ΔHDX (%) summed across all time points. Coloring is based on DynamX residue-level scripts; no statistical filters are applied. Residues without coverage are in gray. **(D)** shows the *Hs* octamer from PDB 2CV5, **(E)** shows the *Xl* octamer from PDB 1AOI, and **(F)** shows the H2A.Z variant octamer from PDB 3WA9.

**
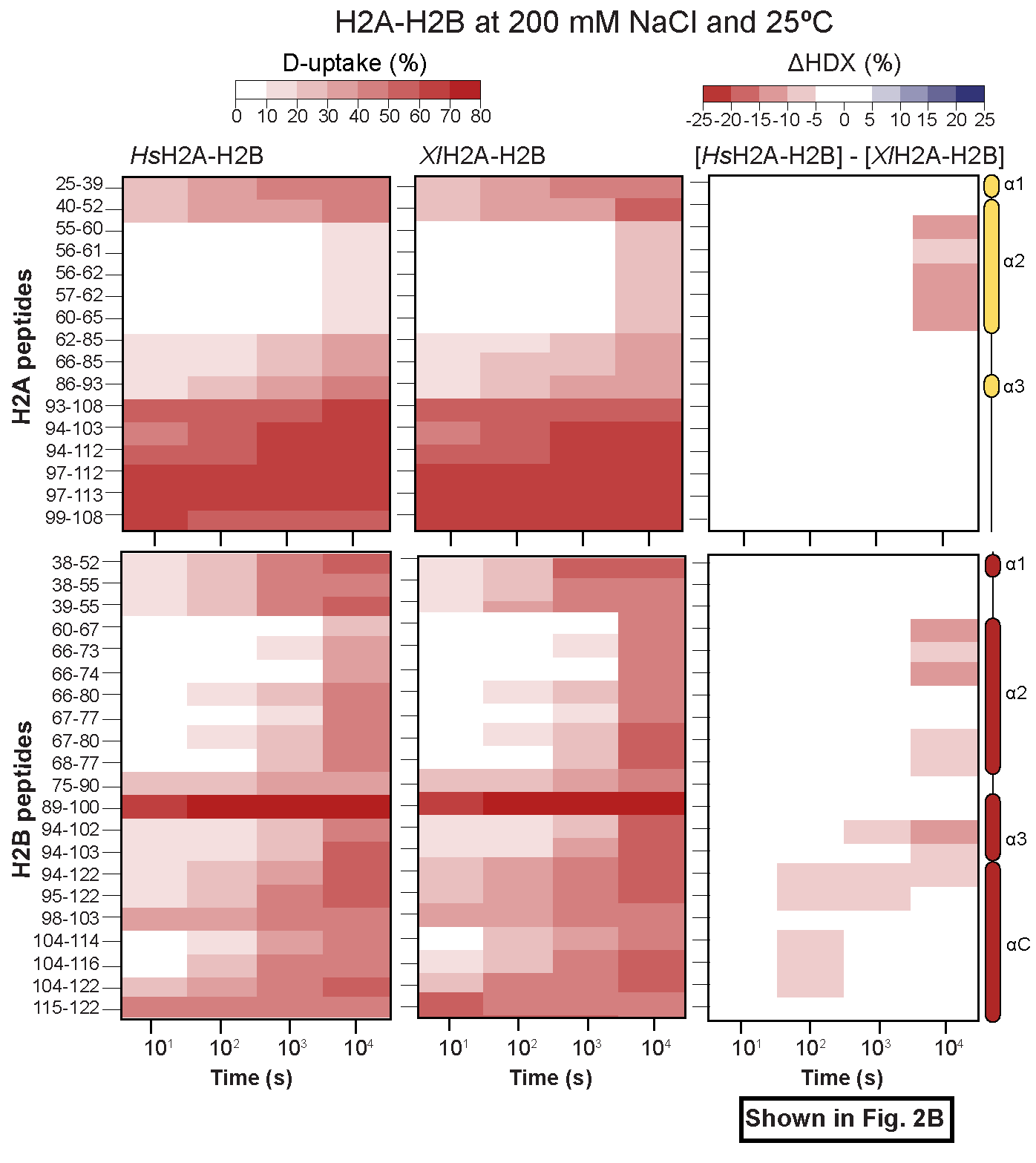
**

**Figure S2: Comparison of *Hs* and *Xl*H2A-H2B at 200 mM NaCl and 25^o^C.** *Left* panel shows deuterium uptake (%) for *Hs*H2A-H2B. *Middle* panel shows deuterium uptake (%) for *Xl*H2A-H2B. *Right* panel shows the deuterium uptake difference (ΔHDX) between the *Hs*H2A-H2B and *Xl*H2A-H2B. Differences are ≥5% and have a *p*-value <0.01 in Welch’s t-test (n=3). Histone α-helices are shown as yellow (H2A) or red (H2B) cylinders.

**
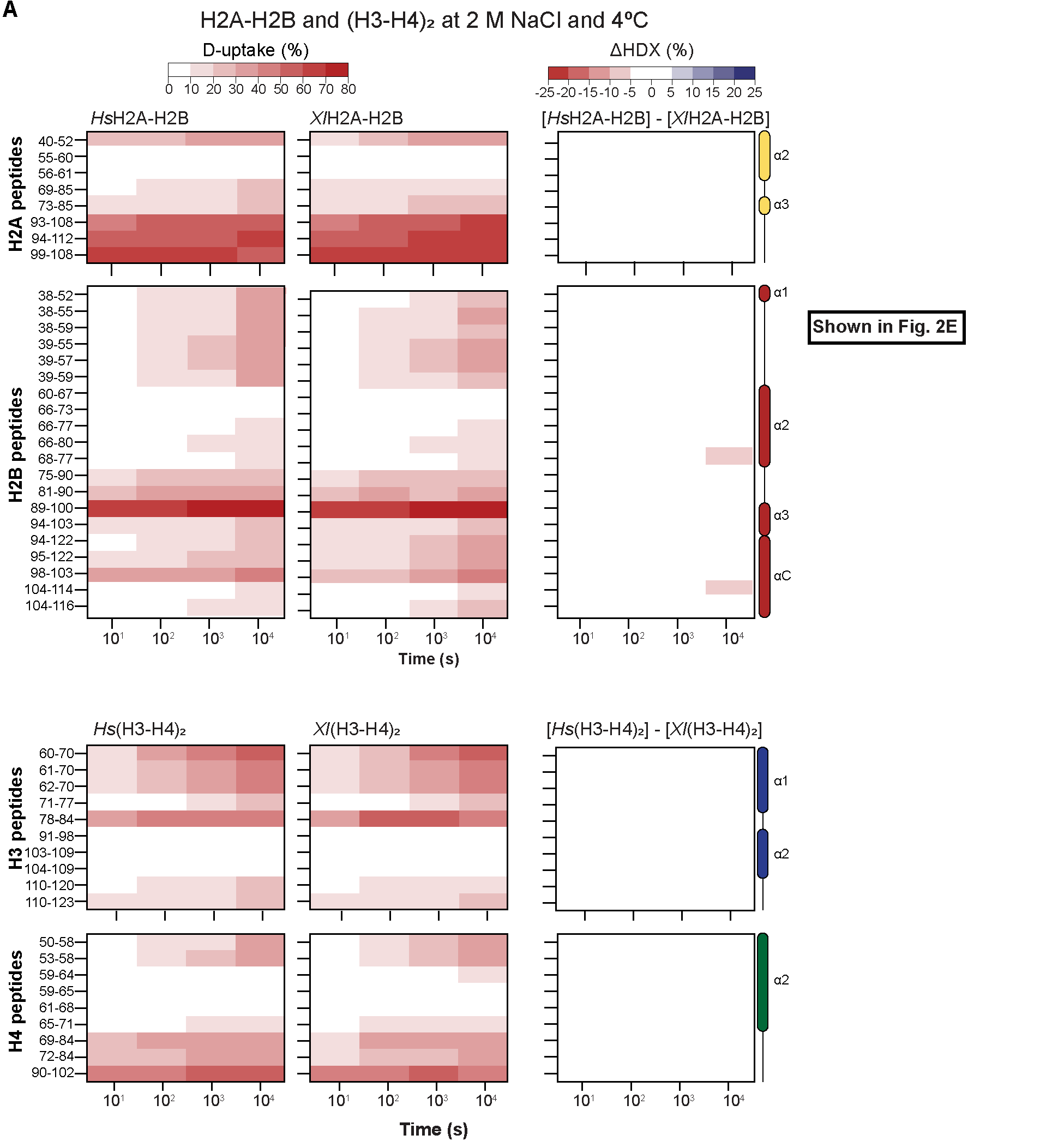

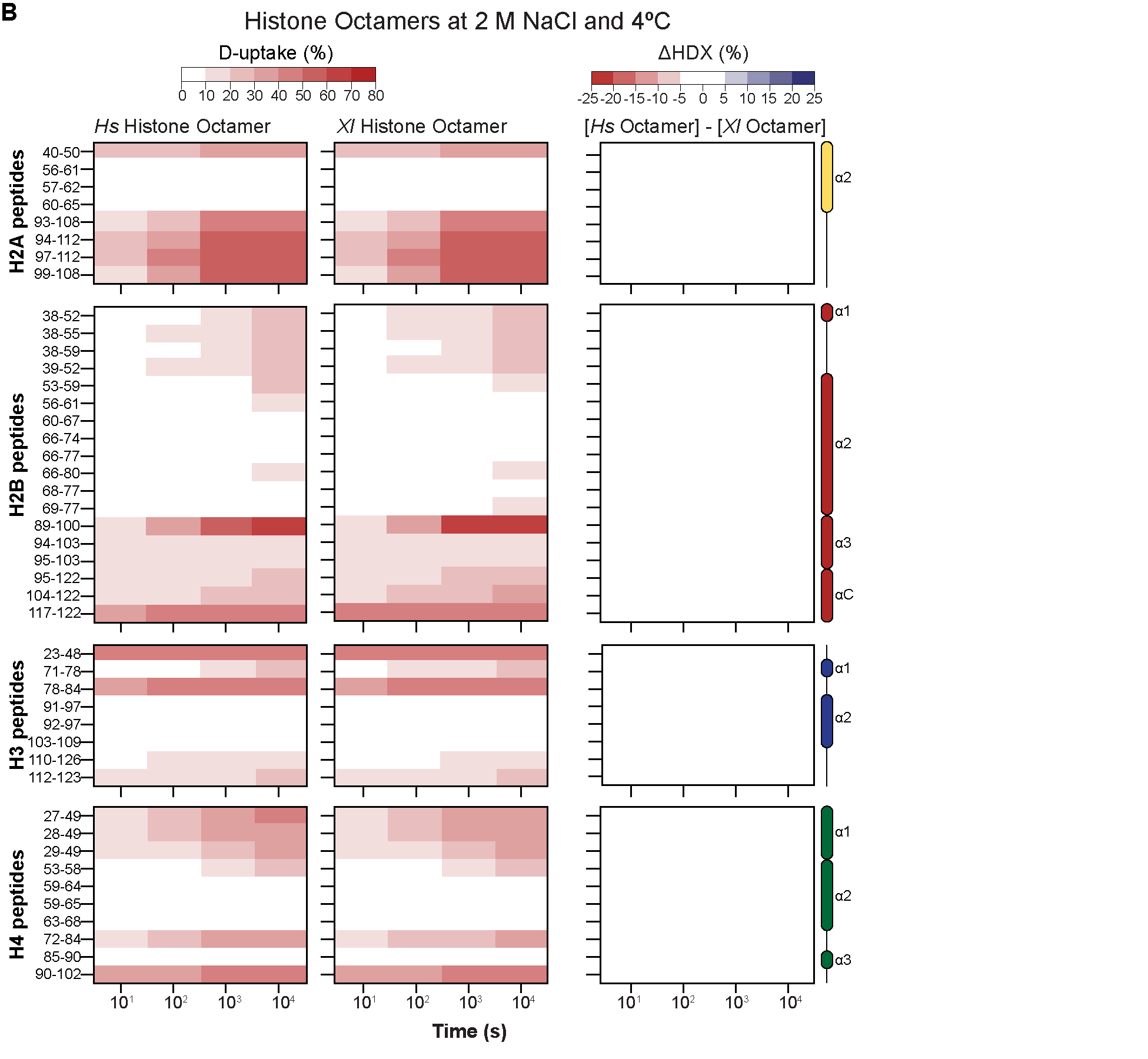
Figure S3: Dynamic changes between *Xl* and *Hs* free histones (A) and histone octamers (B) at 2 M NaCl and 4^o^C.** **(A-B)** *Left* panel shows deuterium uptake (%) for the *Hs* histones. *Middle* panel shows deuterium uptake (%) for *Xl* histones. *Right* panel shows the deuterium uptake difference (ΔHDX) between the *Xl* histones and the *Hs* histones. Differences are ≥5% and have a *p*-value <0.01 in Welch’s t-test (n=3). Histone α-helices are shown as yellow (H2A), red (H2B), blue (H3), or green (H4) cylinders.

**
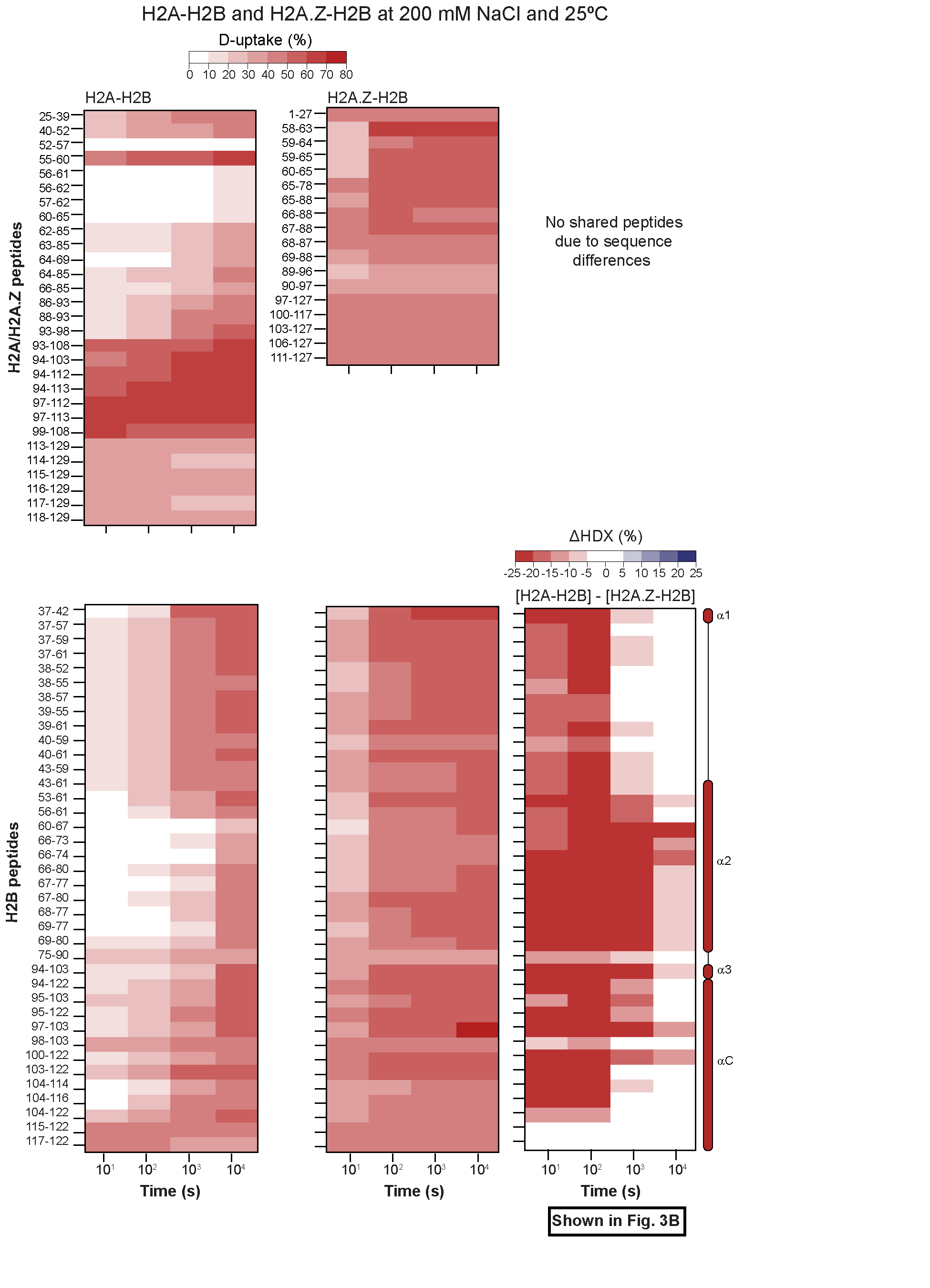
Figure S4: Comparison of core H2A-H2B and variant H2A.Z-H2B at 200 mM NaCl and 25^o^C.** *Left* panel shows deuterium uptake (%) for H2A-H2B. *Middle* panel shows deuterium uptake (%) for H2A.Z-H2B. *Right* panel shows the deuterium uptake difference (ΔHDX) between the H2A-H2B and H2A.Z-H2B. Differences are ≥5% and have a *p*-value <0.01 in Welch’s t-test (n=3). H2B α-helices are shown in red cylinders.

**
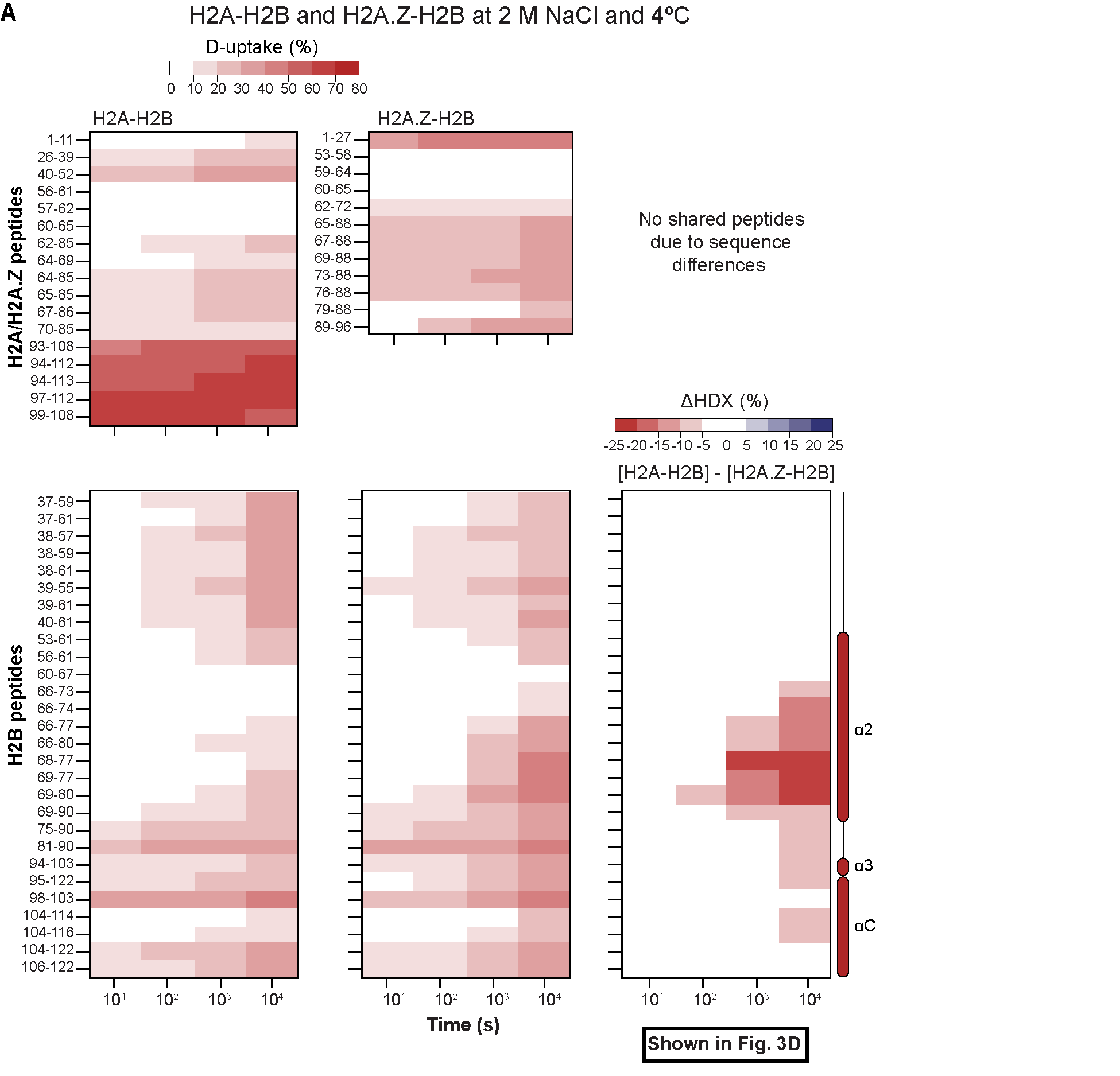

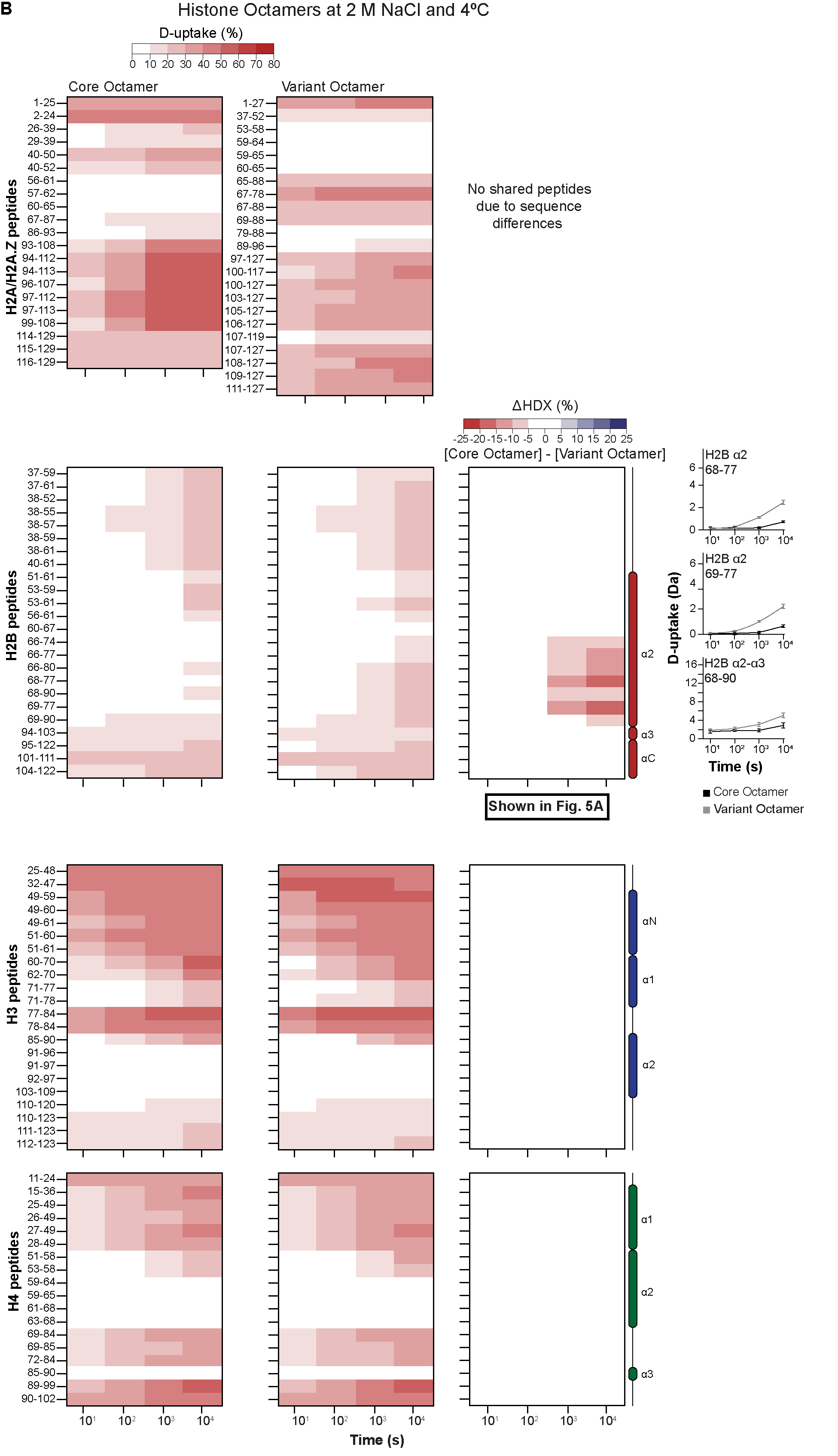
**

**Figure S5: Dynamic changes between core and variant free histones (A) and histone octamers (B) at 2 M NaCl and 4^o^C.** **(A-B)** *Left* panel shows deuterium uptake (%) for the core histones. *Middle* panel shows deuterium uptake (%) for variant histones. *Right* panel shows the deuterium uptake difference (ΔHDX) between the core histones and the variant histones. Differences are ≥5% and have a *p*-value <0.01 in Welch’s t-test (n=3). Histone α-helices are shown as red (H2B), blue (H3), or green (H4) cylinders. In (B), example deuterium uptake (D-uptake) plots for H2B peptides have core octamer in black, and variant octamer in gray. The y-axis is 80% of the maximum theoretical D-uptake. Error bars are ±2 SD with n=3 or 4.

**
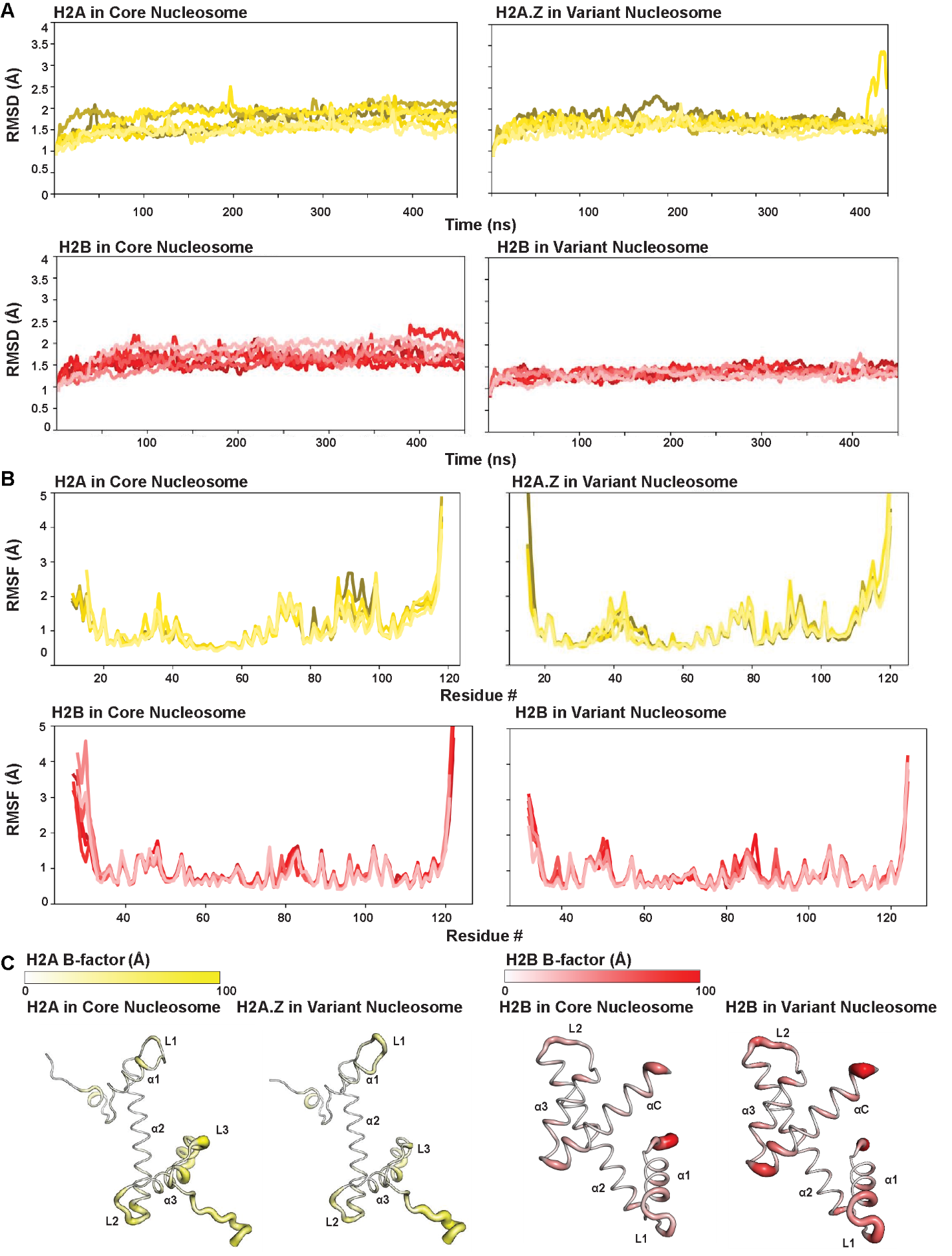
**

**Figure S6: RMSD (A), RMSF (B), and B-factor (C) analysis of H2A-H2B and H2A.Z-H2B in core and variant nucleosomes, respectively. (A-B)** H2A replicates are shown in shades of yellow. H2B replicates are shown in shades of red. **(C)** The thickness of the cartoon (tube) representation and intensity of the colors correspond to B-factor values derived from average RMSF. Thicker and darker regions indicate higher flexibility, while thinner and lighter regions represent more rigid areas.

**A**
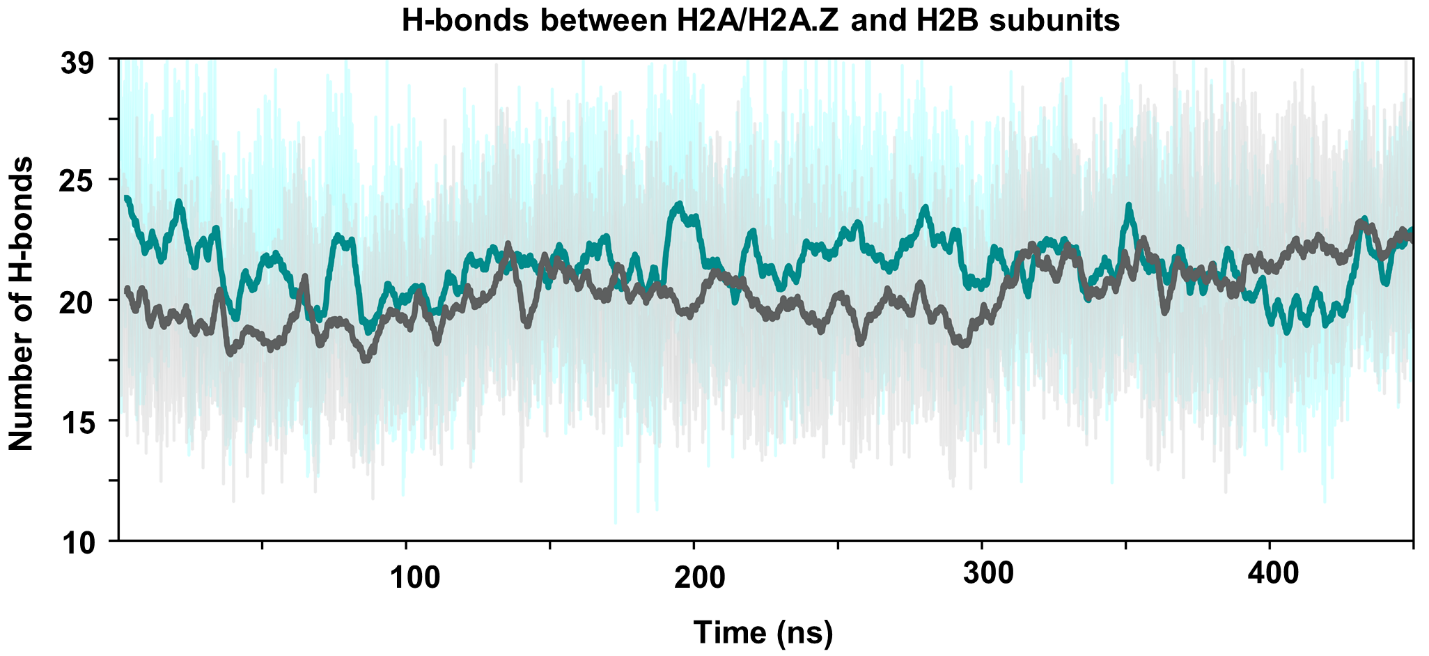


**B**


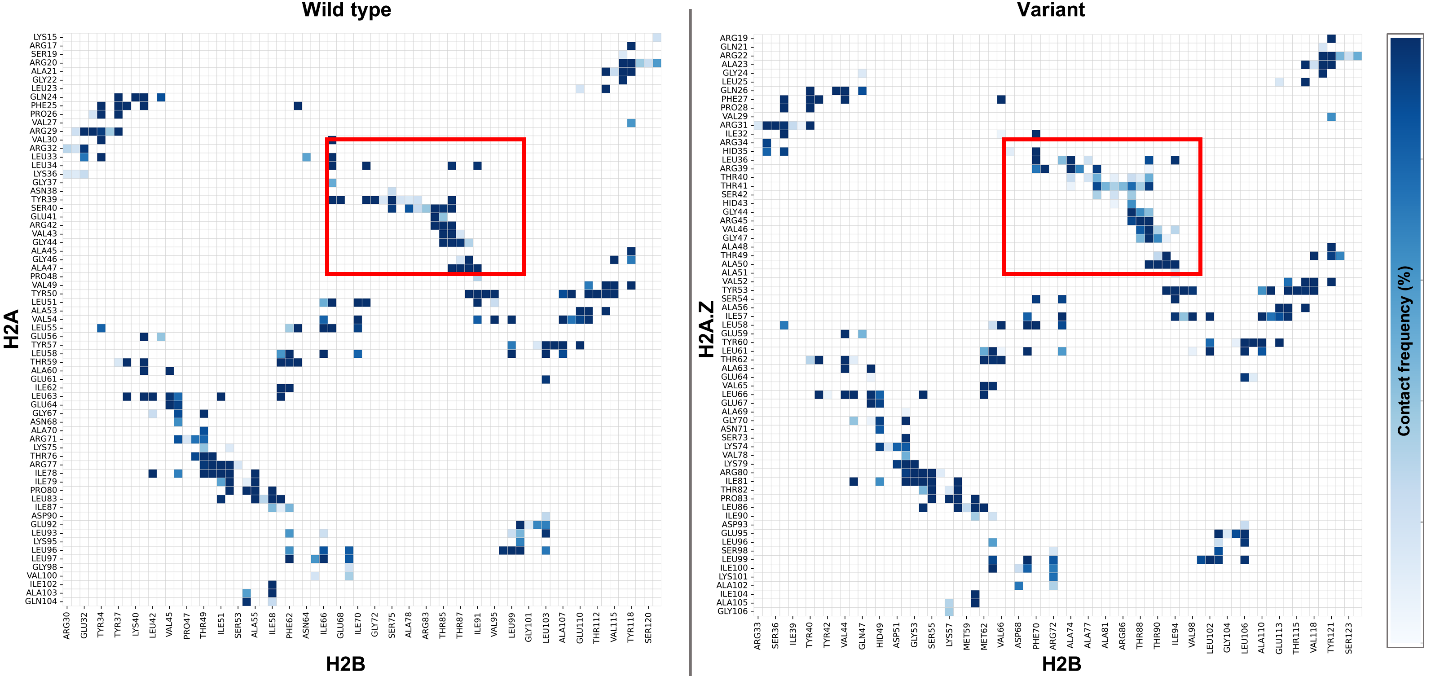


**Figure S7: Hydrogen bonds (H-bonds) between H2A or H2A.Z and H2B in a nucleosome. (A)** Average number of hydrogen bonds formed between H2B and H2A (teal) or H2A.Z (grey) in the nucleosome. **(B)** Contact frequency heatmaps between H2B and H2A (*left*) or H2A.Z (*right*) in the nucleosome. The y-axis represents residues of H2A (*left*) or H2A.Z (*right*), and the x-axis represents residues of H2B. Each square indicates the frequency of contact (defined as within 4.5 Å) observed during the MD simulations, averaged over all replicates. The intensity of the color indicates the percentage contact frequency (0 to 100%). The highlighted (red) regions correspond to the L1 loop of H2A/H2A.Z and the α2–L2 region of H2B, where contact frequency is reduced and dispersed in the variant compared to the core.
